# Biorenewable Solvents for High-Performance Organic Solar Cells

**DOI:** 10.1021/acsenergylett.3c00891

**Published:** 2023-06-16

**Authors:** Julianna Panidi, Eva Mazzolini, Flurin Eisner, Yuang Fu, Francesco Furlan, Zhuoran Qiao, Martina Rimmele, Zhe Li, Xinhui Lu, Jenny Nelson, James R. Durrant, Martin Heeney, Nicola Gasparini

**Affiliations:** † Department of Chemistry & Centre for Processable Electronics, Imperial College London, London W12 0BZ, U.K.; ‡ School of Engineering and Materials Science (SEMS), Queen Mary University of London, London E1 4NS, U.K.; § Department of Physics & Centre for Processable Electronics, Imperial College London, London W12 0BZ, U.K.; ∥ Department of Physics, The Chinese University of Hong Kong, Shatin, Hong Kong SAR 999077, People’s Republic of China; ⊥ King Abdullah University of Science and Technology (KAUST), KAUST Solar Center (KSC), Physical Sciences and Engineering Division (PSE), Thuwal 23955-6900, Saudi Arabia; # Department of Materials Science and Engineering and SPECIFIC IKC, Swansea University, Bay Campus, Fabian Way, Swansea, Wales SA1 8EN, U.K.

## Abstract

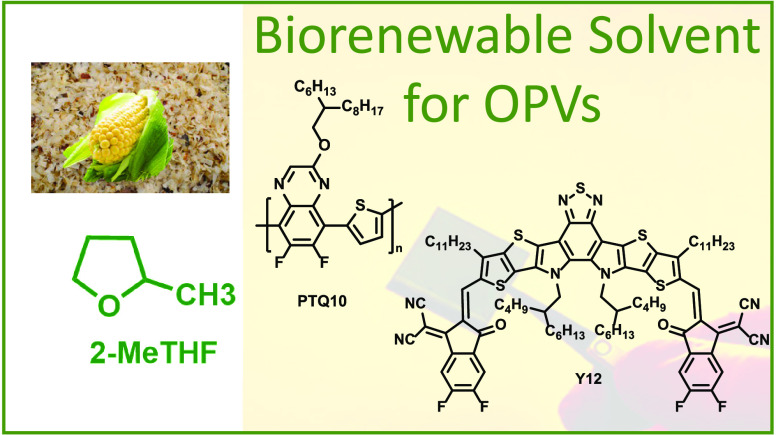

With the advent of nonfullerene acceptors (NFAs), organic photovoltaic (OPV) devices are now achieving high enough power conversion efficiencies (PCEs) for commercialization. However, these high performances rely on active layers processed from petroleum-based and toxic solvents, which are undesirable for mass manufacturing. Here, we demonstrate the use of biorenewable 2-methyltetrahydrofuran (2MeTHF) and cyclopentyl methyl ether (CPME) solvents to process donor: NFA-based OPVs with no additional additives in the active layer. Furthermore, to reduce the overall carbon footprint of the manufacturing cycle of the OPVs, we use polymeric donors that require a few synthetic steps for their synthesis, namely, PTQ10 and FO6-T, which are blended with the Y-series NFA Y12. High performance was achieved using 2MeTHF as the processing solvent, reaching PCEs of 14.5% and 11.4% for PTQ10:Y12 and FO6-T:Y12 blends, respectively. This work demonstrates the potential of using biorenewable solvents without additives for the processing of OPV active layers, opening the door to large-scale and green manufacturing of organic solar cells.

Organic photovoltaics (OPVs) have shown great progress in the past few years owing to the development of nonfullerene acceptors (NFAs) and have recently achieved a power conversion efficiency (PCE) higher than 19%.^1,2^ This technology is fast approaching readiness for wider market commercialization, with a predicted compound annual growth rate of 12.3% between 2020 and 2027.^3^ The excellent performance of OPVs in indoor settings has enabled the realization of unique applications, such as their use as power sources for wirelessly powered sensor nodes.^4,5^

Compared to existing technology such as silicon, solution-processed organic solar cells have the potential for rapid large-scale manufacturing using inexpensive equipment and sustainable materials, leading to extremely short energy and cost payback times.^6,7^ However, realizing this potential requires reducing hazardous chemicals and materials used during fabrication processes and utilizing low-energy consumption techniques for their development.^8^ A major technological challenge to achieve this is by replacing toxic or non-sustainable solvents in the deposition process of the photoactive organic thin films.^9^ This will enable faster translation from the laboratory to mass manufacturing facilities, lower manufacturing costs, enable safer workplaces, and will result in more sustainable end products.

Currently, chlorinated solvents are the most used solvent type in the organic photovoltaic community, as they enable the highest efficiency in devices due to their optimal ability to solubilize organic semiconductors^9^ and offer optimal morphology. The boiling point and the vapor pressure of the solvent have also an effect on the morphology of the donor:acceptor blend, as well as in the film’s surface roughness and the donor:acceptor miscibility.^10^ For instance, a slow evaporation rate will result in bigger aggregates which may be detrimental for OPVs.^11^ However, chlorinated solvents are also among the most toxic and environmentally damaging solvents,^12^ and there has therefore been considerable research into fabricating organic photoactive layers for high-efficiency OPV devices using “greener” nonchlorinated solvents. In particular, excellent results have been achieved with the use of nonhalogenated aromatic solvents such as 1,2-xylene and toluene, with the highest achieved power conversion efficiency (PCE) of 18.52% using 1,2-xylene approaching that of the best devices deposited from chlorinated solvents.^13^ However, aromatic solvents are petroleum-based, which are associated with numerous hazards during solvent production^14^ and use in large quantities, which raises significant questions over their suitability as nontoxic and “green” solvents for large-scale OPVs and organic electronics manufacturing.^15,16^ For example, terpene-based solvents have recently been reported as a great alternative for OPVs active layers; however, for optimal OPV performance, a significant (>50%) proportion of aromatic solvent was still required in the total solvent mixtures.^17^ Another suggestion for green solvents has been tetrahydrofuran (THF) and 2-methyltetrahydrofuran (2MeTHF), which have been investigated in all-polymer OPVs (Table S1).^18−20^ Thus, there is still an urgent need for enabling the fabrication of high-efficiency organic solar cells using exclusively nonaromatic solvents.

The choice of a “green” solvent is nontrivial due to various, potentially competing factors that can be important. These include safety (flammability and flash point, peroxide risk), health (acute, short-term, and long-term toxicity), environment (ecotoxicity, life cycle analysis, biodegradability, sustainability), and cost. Various guides have been produced, especially by chemical companies, to help guide the selection and potential replacement of undesirable solvents.^21,22^ Based on these guides, we identified 2MeTHF and cyclopentyl methyl ether (CPME) as potential candidates of interest, particularly since the former is now commercially available from biorenewable sources. Biorenewable 2MeTHF is derived from furfural or levulinic acid, whilst^23,24^ CPME is currently petroleum-based but can be prepared from biorenewable sources via the addition of methanol to cyclopentene or the methylation of cyclopentanol from biomass-based adipic acid or furfural, respectively. Moreover, CPME is considered a greener alternative to many aprotic ether solvents, better resisting the formation of potentially explosive peroxides and requiring less energy during solvent production.^25,26^ Inevitably they are not the perfect solvents, and some compromises must be made, but we note that Pfizer and Sanofi denote 2MeTHF as usable and preferred, respectively, while GSK highlights “some issues”. These issues are primarily related to the flammability of the solvent and its propensity to form peroxides. The latter is reduced for CPME, prompting our investigation, and we also investigated both inhibited and inhibitor-free grades of 2MeTHF, with the former reducing the peroxide formation risk. Both solvents compare favorably to 1,2-xylene, a common nonhalogenated aromatic solvent derived from oil-based feedstocks, according to our analysis based on safety data sheets (see Table S2) and the published solvent guides.

Herein, we report the development of high-performance organic solar cells based on 2METHF and CPME , without the need for toxic additives and using low-synthetic-cost polymer donors. We investigate two synthetically simple polymeric donors, PTQ10^27^ and FO6-T, blended with the nonfullerene acceptor BTF-4F-12 (Y12). We achieve promising PCEs of over 14% and 11% with PTQ10:Y12 and FO6-T:Y12 processed from 2MeTHF, respectively, due to the optimal blend morphology and molecular orientations in thin films deposited using this solvent. In contrast, we find a much lower performance in blends processed from CPME due to the much higher crystallinity of the deposited thin films with suboptimal mixed face-on and edge-on orientations. Finally, we also demonstrate OPVs with active layers processed in air using blade-coating, delivering PCEs of 13.8% and 12% for PTQ10:Y12 and FO6-T:Y12, respectively, using 2MeTHF.

The PM6:Y6 blend has changed the landscape of OPV devices, with high efficiency being reported by many research groups. Despite these great advances, PM6 has very low solubility (<0.5 mg/mL) in both 2MeTHF and CPME solvents (Figure S1), as well as in terpene-based solvents, as recently reported by Corzo et al.^17^ In addition, PM6 has a high degree of synthetic complexity and requires 15 synthetic steps to produce.^28^ The donor materials PTQ10^29^ and FO6-T, used in this study, require significantly less steps for their preparation, which is aligned with our efforts for high-performance sustainable organic solar cells. Reducing the synthetic complexity is highly important for facilitating upscaling, as well as reducing the overall energy and water requirements during synthesis, which in turn reduces the carbon footprint of the final device. Additionally, the solubility of these polymers in the biosourced solvents is much higher than that of PM6, with FO6-T having a solubility limit of around 12 and PTQ10 of 4.6 mg/mL (Figures S2–S5 and Table S3) in 2MeTHF at room temperature, while PM6 did not dissolve at all in either solvent. For the acceptor, we chose the Y6 derivate Y12, with longer side chains optimized for solubility in nonchlorinated solvents.^30^ Similarly to the polymers, Y12 showed a higher solubility (6.7 mg/mL) in 2MeTHF compared to CPME (1.5 mg/mL) at room temperature. We also evaluated the interaction parameter χ between the donor and acceptor materials processed using different solvents from contact angle measurements. From all measurements (Figure S6) it can be seen that PTQ10 has slightly better miscibility with Y12 (in 2MeTHF χ is 0.33) than FO6-T with Y12 (in 2MeTHF χ is 0.55) in the three tested solvents (Table S4).

The chemical structures and energy levels of the organic semiconductors used in this study are shown in Figure 1a,b. The highest occupied molecular orbital (HOMO) was measured using air photoemission spectroscopy of thin films deposited from 2MeTHF on ITO glass substrates. The LUMO energy level was estimated by adding the optical band gap to the HOMO level. Absorption measurements (UV–vis) were carried out on the pristine polymers and NFA (Figure S7) to extract their optical band gap in 2MeTHF, CPME, and 1,2-xylene. 1,2-Xylene was used as the reference solvent for these systems because of its high performance in both Y12-based and PTQ10-based organic solar cells.^31^ The HOMO levels of PTQ10 and FO6-T were measured as −5.18 and −5.08 eV, respectively, whereas the LUMO of Y12 is calculated at −4.46 eV, suggesting good energetic alignment between the donor and acceptor material.

**Figure 1:**
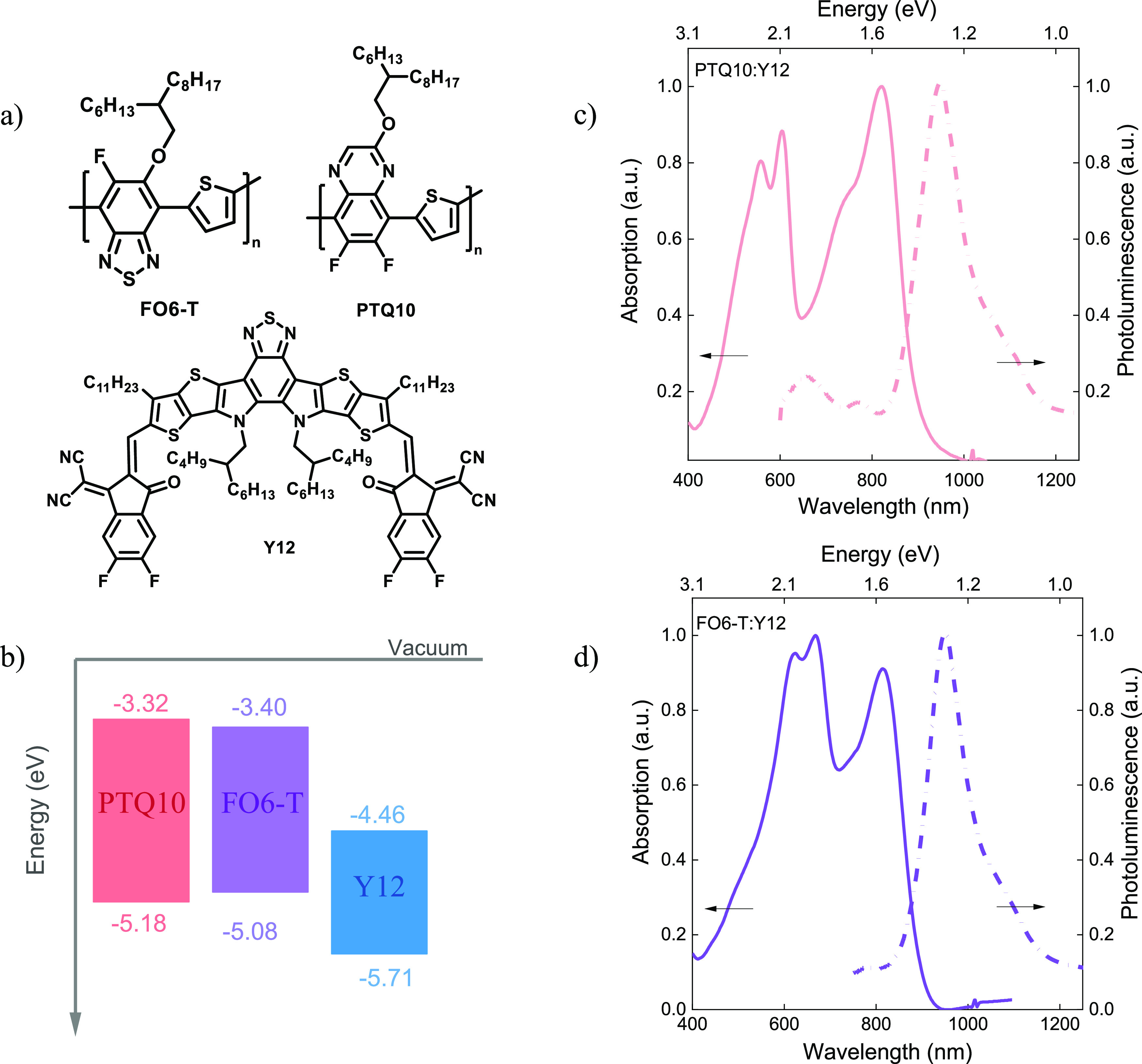
(a) Chemical structures of FO6-T and PTQ10 donors and Y12 acceptor. (b) Energy level diagram of the materials used. The HOMO level was measured by air photoemission spectroscopy, and the LUMO level was estimated from the optical band gap. UV–vis and PL spectra of (c) PTQ10:Y12 and (d) FO6-T:Y12 blends processed from 2MeTHF.

The UV–vis and photoluminescent (PL) spectra of PTQ10:Y12 and FO6-T:Y12 thin film blends processed from 2MeTHF are shown in Figures 1c,d, respectively. As expected, Y12 has strong absorption from 600 to 980 nm, with a peak PL emission at around 970–980 nm in films processed from all solvents. Interestingly, Y12 processed from 1,2-xylene and 2MeTHF shows almost identical absorption and emission, suggesting very similar film aggregation properties.^32^ In contrast, Y12 processed from CPME has slightly blue-shifted absorption and red-shifted photoluminescence peaks, with a strong luminescence shoulder peak at 1050 nm, suggesting different aggregation properties of Y12 processed from CPME than from 2MeTHF and 1,2-xylene.

Among the polymers, PTQ10 showed strong absorption and emission at around 420–730 and 620–900 nm, respectively, with little change in the spectra between different solvents, while the spectra of FO6-T are red-shifted by ∼100 nm compared to PTQ10 owing to its smaller band gap. In blends, for PTQ10:Y12 the absorption spectra from CPME and 2MeTHF are broadly similar, comprising strong absorption of PTQ10 at around 420–730 nm and Y12 absorption at around 600–980 nm, while in 1,2-xylene the absorption of PTQ10 appears slightly weaker than in the blends processed from the other solvents. The relative strengths of the vibronic peaks of both Y12 and PTQ10 in the blends were similar to those of the pristine materials, suggesting that the microstructure of the pristine materials was broadly retained in the pure phases of the blend films processed from all solvents.

A similar analysis for PTQ10:Y12 holds for FO6-T:Y12 blends processed from 1,2-xylene and 2MeTHF; however, for blends processed from CPME, the spectra are dominated by donor absorption and emission with only a low signal contribution from Y12, suggesting that CPME adversely affects the blend morphology formation in this blend. This is in agreement with the much lower PL quenching observed in the CPME-processed FO6-T:Y12 films compared with all other blends (Figure S8), which may suggest strong phase separation leading to poor exciton dissociation in this film.

PTQ10:Y12 and FO6-T:Y12 OPV devices were fabricated from 2MeTHF, CPME, and 1,2-xylene (as a reference) in an inverted structure, and representative *J*–*V* curves measured under AM1.5G illumination at 100 mW/cm^2^ are shown in Figure 2. The highest previously reported PCE obtained from 1,2-xylene was 11.3% for PTQ10:Y12 OPVs.^27^ Here, for both blend systems, 2MeTHF seems to be an ideal solvent for processing the active layer, offering the best performance among the three solvents, reaching a high PCE of 14.5% for PTQ10:Y12 with a short circuit current density (*J*_sc_) of 24.6 mA/cm^2^, an open circuit voltage (*V*_oc_) of 0.86 V and a fill factor (FF) of 0.68. A slightly lower performance was achieved with 1,2-xylene-based OPVs with a PCE of 12.4%, primarily due to a lower *J*_sc_ of 21.3 mA/cm^2^. The lowest overall performance was obtained when PTQ10:Y12 was processed using CPME, reaching a PCE of 4.9%.

**Figure 2:**
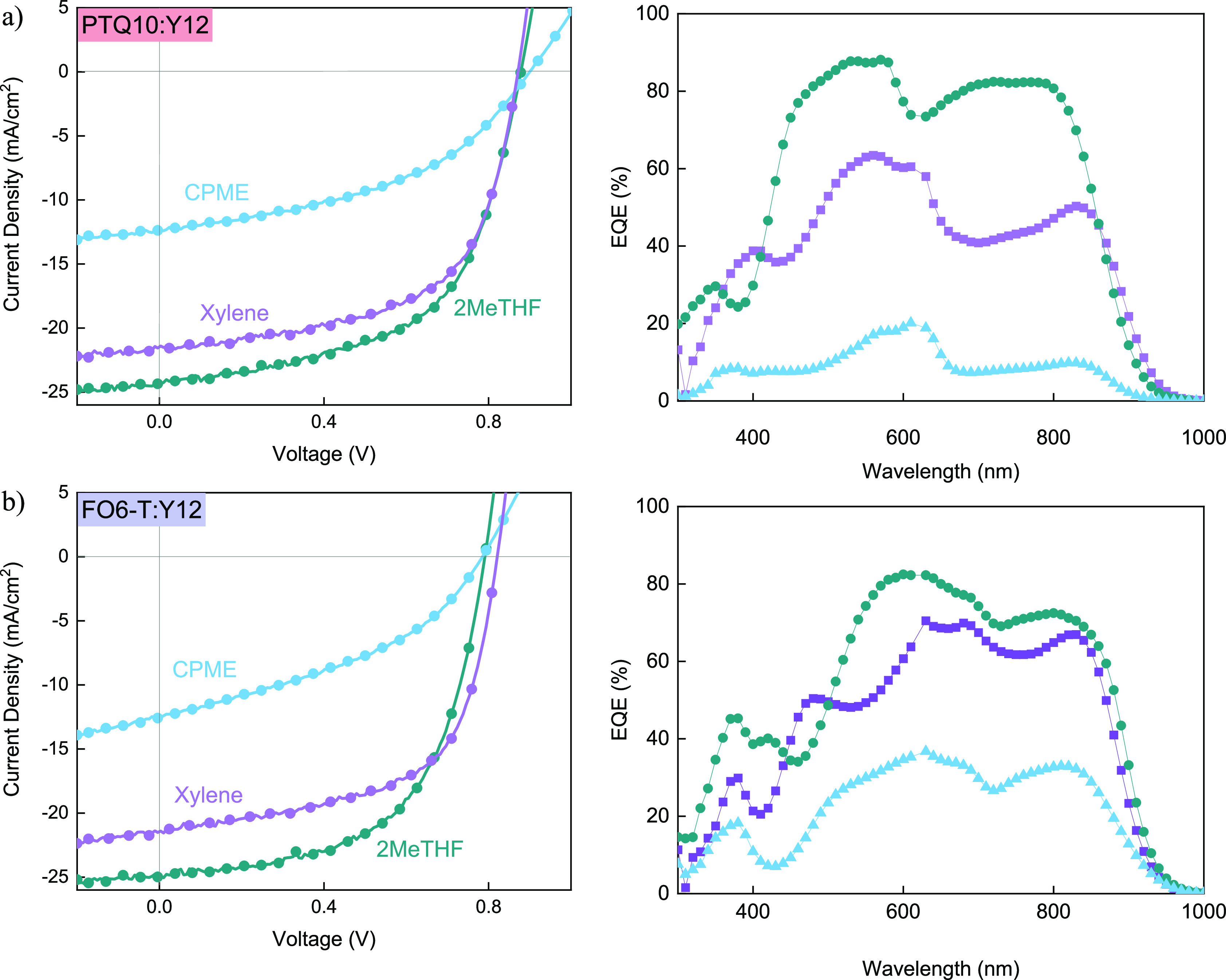
Representative *J*–*V* and EQE characteristics for (a) PTQ10:Y12- and (b) FO6-T:Y12-based OPVs when the active layer was processed from 2MeTHF, CPME, and 1,2-xylene.

A similar trend was observed for the FO6-T:Y12 (Figure 2b) blend, with the highest PCE, reaching 11.4% when the active layer was processed from 2MeTHF, with a *J*_sc_ of 26.3 mA/cm^2^, a *V*_oc_ of 0.79 V, and a FF of 0.58. The 1,2-xylene-based OPVs showed a slightly lower PCE of 10.8% with a *V*_oc_ of 0.82 V, a FF of 0.61, and a *J*_sc_ of 21.3 mA/cm^2^. For the CPME-processed FO6-T:Y12 based OPVs, a low efficiency of 3.3% was achieved with significantly reduced FF and *J*_sc_ values. All device parameter statistics are given in Table 1. Similar performance was obtained when OPVs were prepared in a normal device architecture, reaching PCEs of 14% and 10.8% for the PTQ10:Y12 and FO6-T:Y12 blends, respectively, as presented in Figure S9 and in the summary results in Table S5.

**Table 1:** OPV Parameters for PTQ10:Y12 and FO6-T:Y12 Devices When the Active Layer Was Processed from 1,2-Xylene, 2MeTHF, and CPME showing the Mean, Standard Deviation, and Maximum Value (Shown in Parentheses) from 18 Devices

	*J*_sc_ (mA/cm^2^)	*V*_oc_ (V)	FF	PCE (%)
PTQ10:Y12				
1,2-xylene	20.03 ± 0.92 (21.3)	0.87 ± 0.01 (0.87)	0.61 ± 0.01 (0.60)	10.74 ± 0.50 (12.4)
2MeTHF	23.41 ± 0.60 (24.6)	0.85 ± 0.01 (0.86)	0.66 ± 0.01 (0.68)	13.37 ± 0.51 (14.5)
CPME	10.82 ± 1.52 (12.3)	0.80 ± 0.16 (0.89)	0.38 ± 0.04 (0.44)	3.25 ± 1.16 (4.9)
FO6-T:Y12				
1,2-xylene	20.16 ± 0.88 (21.3)	0.78 ± 0.07 (0.82)	0.59 ± 0.06 (0.61)	9.52 ± 1.07 (10.8)
2MeTHF	25.00 ± 0.85 (26.3)	0.77 ± 0.01 (0.79)	0.55 ± 0.01 (0.58)	11.01 ± 0.46 (11.4)
CPME	9.84 ± 1.26 (11.1)	0.68 ± 0.07 (0.79)	0.37 ± 0.01(0.36)	2.44 ± 0.65 (3.3)

The respective external quantum efficiency (EQE) spectra of the PTQ10:Y12 and FO6-T:Y12 blends in 2MeTHF and 1,2-xylene OPV cells are presented in Figure 2, showing good current generation across the entire absorption range of the blends for all devices. The integrated current density, as extracted from the EQE spectra (Figure S10), is well aligned with the values obtained from the *J*–*V* measurements. A maximum EQE of 88% was obtained from the 2MeTHF-processed PTQ10:Y12 devices and 82% from FO6-T:Y12, while for the 1,2-xylene based OPVs, an EQE of 64% was obtained from PTQ10:Y12 and 70% from FO6-T:Y12. These results show that high photovoltaic performance can be maintained by replacing halogenated and aromatic solvents with next-generation eco-friendly alternatives and synthetically simple donor polymers. Finally, the stability of the 2MeTHF-processed devices was evaluated for 1000 h under 1 sun illumination (LED spectrum, Figure S12) under nitrogen conditions (not encapsulated devices) of continuous testing at the maximum power point (Figure S11). PTQ10:Y12 OPVs show a 20% reduction after around 350 h, whereas the performance of FO6-T:Y12 devices stable across 1000 h.

The charge carrier mobilities of the organic semiconducting blends used as OPV active layers were extracted via the space charge limited current method.^33^ One-carrier type devices were fabricated to evaluate the electron (μ_e_) and hole (μ_h_) transport y and the *J*–*V* characteristics are presented in Figures S13 and S14. For both 1,2-xylene- and 2MeTHF-processed active layers, we calculated similarly high and relatively balanced charge carrier mobilities, whereas for the CPME-based device, we find a drop in electron mobility of about 2 orders of magnitude (9 × 10^–7^ cm^2^ V^–1^ s^–1^) compared to 2MeTHF- and 1,2-xylene-based blends, which leads to unbalanced charge transport, and contributes towards the lower FF in these devices^33^ (Table S6).

A major challenge in further increasing the performance of OPV devices toward that of competing technologies (e.g., silicon, perovskites) is suppressing nonradiative decay pathways in order to decrease voltage losses and therefore increase the achievable open-circuit voltage.^34^ To determine whether using our next-generation solvents places limits on the achievable nonradiative voltage losses of such devices, we further performed a voltage loss analysis of the best-performing 2MeTHF-processed blends using measured electroluminescence (EL) and high-dynamic-range EQE spectra, based on our previously reported approach.^35^ For FO6-T:Y12, we find a relatively high nonradiative voltage (Δ*V*_oc,nrad_) loss of 0.28 V, which agrees with the presence of a low-energy and highly luminescent CT state (see Figure S15) and the relatively large energetic offset between the energy levels of FO6-T and Y12. By contrast, the EL emission of PTQ12:Y12 is completely dominated by Y12 S_1_ emission, leading to a much lower Δ*V*_oc,nrad_ of 0.21 V (Table S7 and Figure S16), in agreement with previous demonstrations of low nonradiative voltage losses when S_1_ and CT-state emission is strongly mixed.^36,37^ Such a low Δ*V*_oc,nrad_ compares extremely favorably with other high-efficiency organic solar cells, using either chlorinated or nonchlorinated solvents,^38^ and suggests that high-quality donor–acceptor interfaces with low CT-state energetic disorder^39,40^ can be achieved using green solvents such as 2MeTHF. We note that achieving such a low Δ*V*_oc,nrad_ is particularly promising given the unique combination of green solvent and synthetically simple polymer that we used here.

To further evaluate the operation mechanism of the best-performing devices when processed from 2MeTHF, we performed light-intensity-dependent *J*–*V* measurements in the range of 5–100 mW/cm^2^. Figure 3a shows the extracted *V*_oc_ dependence of the PTQ10:Y12 and FO6-T:Y12 based OPVs. Both systems show a slope of approximately equal to *kT*/*q*, which is the case for second-order recombination, suggesting that the devices are not suffering from significant trap-mediated bimolecular recombination.^41^ A similar order of the studied systems was also observed for the slope(s) of the *J*_sc_ upon varying the light intensity (Figure S17a), with *s* = 0.9 and 0.88 for the FO6-T:Y12 and PTQ10:Y12 blends, respectively, indicative of some bimolecular recombination losses at short circuit. Surprisingly, the FF of the PTQ10:Y12 devices remains stable throughout the light variation, leading to light-independent device performance, as seen in Figure 3b, which is assigned to reduced traps in the blend.^42^ By contrast, FO6-T:Y12 OPVs show an initial increase in the FF followed by a reduction at higher light intensity values, which can be attributed to increased bimolecular recombination.^43^ We further investigated the photogenerated current density (*J*_ph_) as a function of the effective voltage (*V*_eff_) to evaluate the photocurrent’s influence on the applied voltage at different light intensities,^44^ as shown in Figure S17b. Both systems show a saturated photocurrent with an average value of 26.8 mA/cm^2^ for PTQ10:Y12 and 27.8 mA/cm^2^ for the FO6-T:Y12 blend, leading to similar values of exciton generation rate (*G*_max_) on the order of 1.67 × 10^22^ and 1.73 × 10^22^ cm^–3^ s^–1^, respectively. The light-independent performance of PTQ10:Y12 makes this blend highly versatile for a range of applications, from indoor to concentrated light.

**Figure 3:**
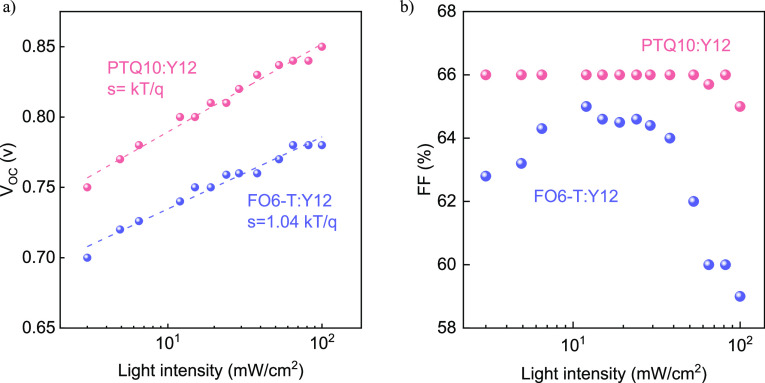
Light-dependent measurements in the range of 5–100 mW/cm^2^ of OPVs from FO6-T:Y12 and PTQ10:Y12 blends processed from 2MeTHF: (a) *J*_sc_ and (b) FF change upon light variation.

When comparing the 1,2-xylene processed OPVs with those processed with 2MeTHF, several differences can be seen from the light-dependence characterization, which might explain the slightly lower performance. 2MeTHF OPVs showed a slightly higher *G*_max_ rate in the saturation regime (Figure S18). The charge carrier generation rate at the maximum power point (*G*_mpp_) shows an achieved rate of 63% for the 1,2-xylene-processed FO6-T:Y12, whereas it is 69% for that processed with 2MeTHF. A similar trend is observed for the PTQ10:Y12 blend, where 55% was achieved with 1,2-xylene and 69% with 2MeTHF. For both 2MeTHF-processed blends, a higher rate was also observed under the short circuit condition (*G*_sc_), when compared with 1,2-xylene processing, as shown in Table S8.

To demonstrate the potential scalability of these materials, PTQ10:Y12 and FO6-T:Y12 devices were fabricated by blade coating in air (Figure 4 and Figure S19). This fabrication process is often used as a proof of concept for larger-area fabrication of organic solar cells, bridging the gap between spin coating and slot-die coating.^45,46^ 2MeTHF was chosen as the processing solvent as a result of its higher performance during spin coating, as discussed above. Very promising PCEs of 13.8% and 12% were obtained for PTQ10:Y12 and FO6-T:Y12, respectively, which are comparable to the performance of the spin-coated devices. In conclusion, these results demonstrate (a) the deposition in air does not have an impact on the OPV performance and (b) doctor-blade deposition results in performance comparable to that of spin-coated OPVs (Table S9).

**Figure 4:**
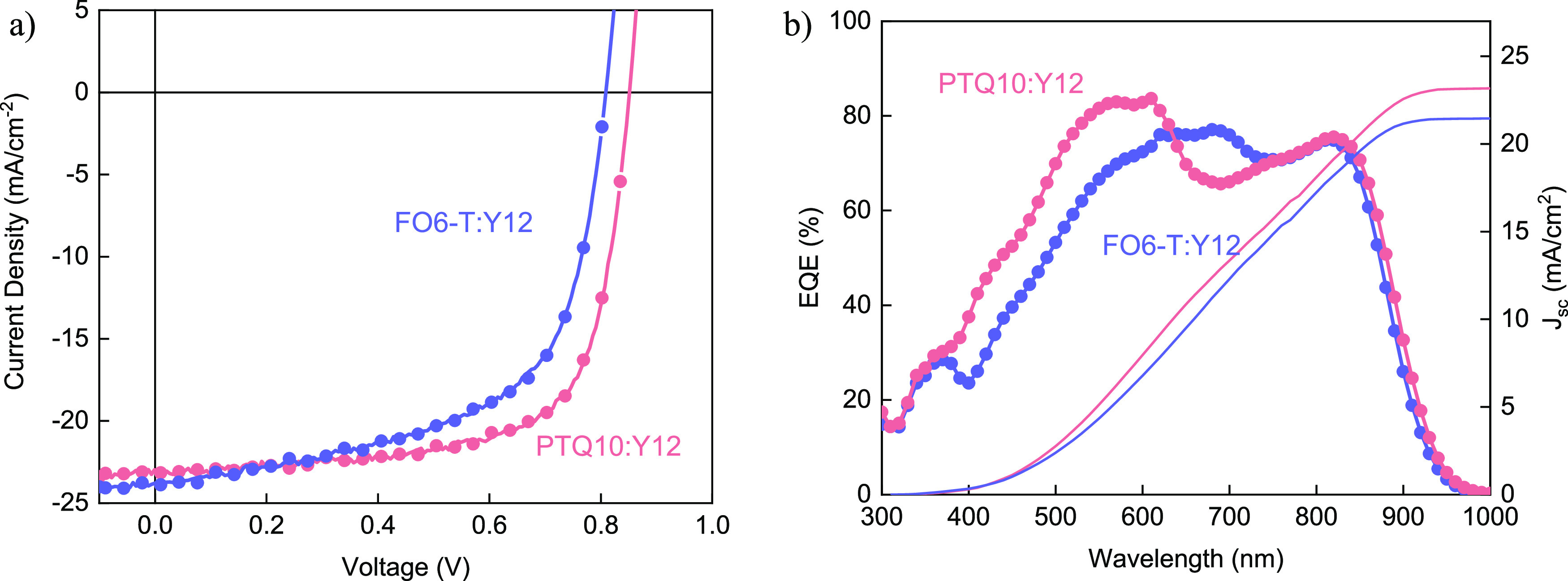
FO6-T:Y12 and PTQ10:Y12 OPVs processed from 2MeTHF via doctor blade: (a) *J*–*V* and (b) EQE representative characteristics.

Finally, to gain a better understanding of the large difference in the performance of OPVs using 2MeTHF and CPME, we performed morphological characterizations of blend and pristine films through grazing incidence wide-angle X-ray scattering (GIWAXS) and grazing incidence small-angle scattering (GISAXS). Figure 5 shows the 2D GIWAXS patterns for FO6-T:Y12 and PTQ10:Y12 in CPME and 2MeTHF. Both blends when processed from 2MeTHF showed a preferred face-on orientation with respect to the substrate, with strong (100) lamellar peaks along the in-plane direction (IP) at 0.30 and 0.27 Å^–1^ for FO6-T:Y12 and PTQ10:Y12, respectively. This peak mainly arises from the donor polymers (Figure S20, for single-component GIWAXS). The *d*_*z*_ spacing was calculated to be 2.1 nm for FO6-T:Y12 and 2.3 nm for PTQ10:Y12. On the other hand, the strong π–π peaks at 1.75 Å^–1^ for FO6-T:Y12 and 1.77 Å^–1^ for PTQ10:Y12 arise from Y12, as can be seen in Figure S20c and Tables S10 and S11. The presence of Y12 molecules in blend films disrupts the polymer’s microstructure, so the higher-order lamellar peaks of observed in polymer neat films are absent in blend films.

**Figure 5:**
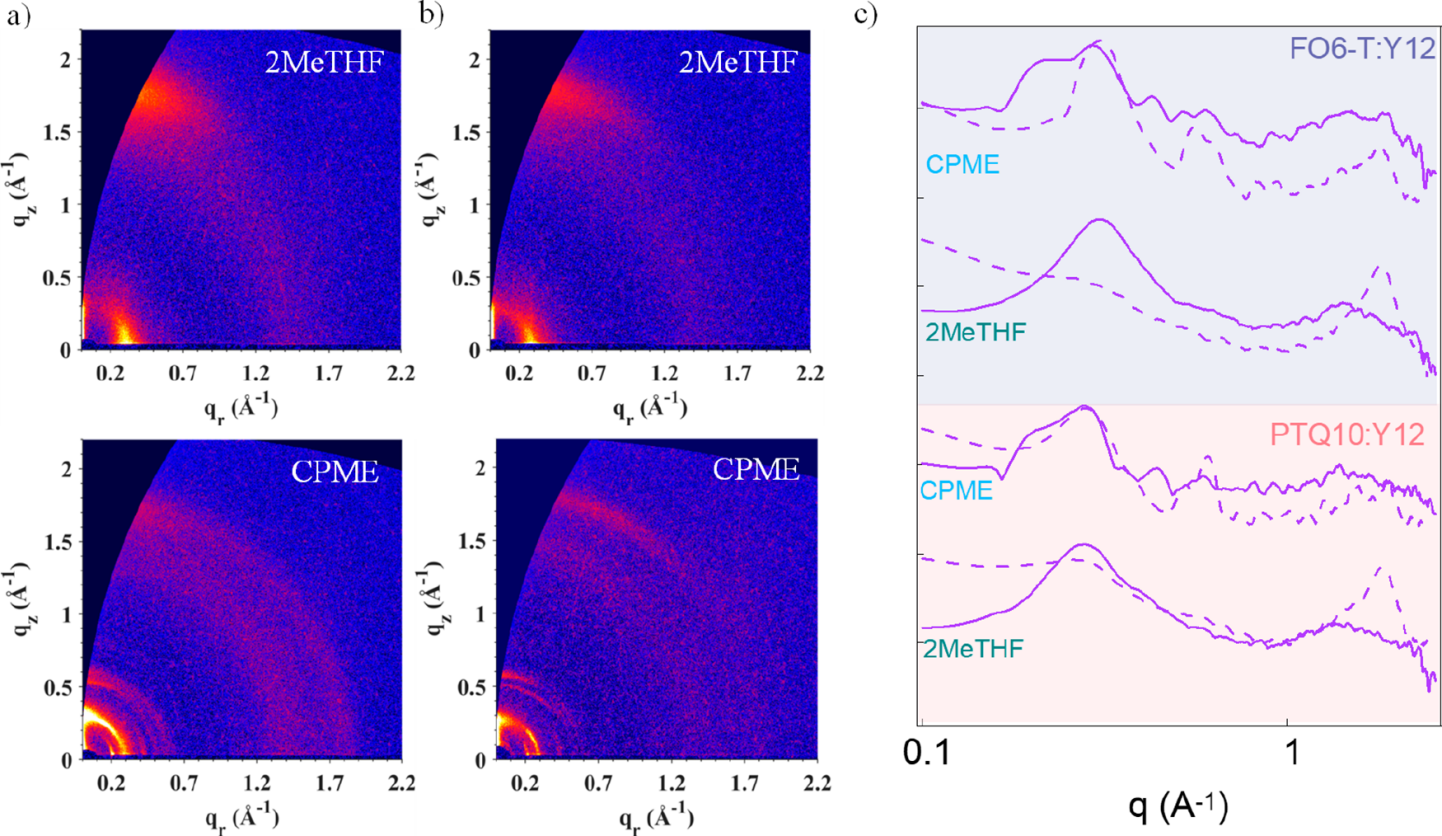
GIWAXS analysis for (a) PTQ10:Y12 and (b) FO6-T:Y12 thin films processed from 2MeTHF and CPME with (c) the respective 2D profiles, with dashed lines representing the line cuts extracted along the in-plane direction and with solid lines extracted along the out-of-plane direction.

When processed from CPME, both PTQ10 and Y12 neat films showed an increased peak intensity and reduced peak width, indicative of a much higher degree of crystallinity and a larger crystal size. For quantitative analysis, the crystalline coherence length (*L*_c_) was calculated using the Scherrer equation, with the peak full width at half-maximum (fwhm) extracted from the Gaussian fitting of the peak. As shown in Tables S10 and S11, the *L*_c_ values of the CPME-processed PTQ10 and Y12 neat films are 1 order of magnitude higher than that of their 2MeTHF-processed counterparts. The large crystallites of PTQ10 and Y12 in the CPME-processed films are retained in blend films. On the other hand, the diffraction pattern of FO6-T remains the same when processed from either CPME or 2MeTHF. Additionally, the molecular orientations became much more random compared to the predominantly face-on orientation in the 2MeTHF-processed films. Both oversized crystallites and random molecular orientations are unfavorable for charge generation and transport,^47−49^ which explains the inferior performance of CPME-processed devices.

The nanomorphology of the blend films was probed via GISAXS, and the average domain size was calculated by the Guinier radius of the fractal-like network *R*_g_.^50^ As shown in Figure S21, when the devices are processed from 2MeTHF, the size of pure domains is much larger in FO6-T:Y12 (51.6 nm) than in PTQ10:Y12 (29.7 nm). Although similar sizes of pure domains of 35.3 and 29.2 nm were obtained for the CPME-processed FO6-T:Y12 and PTQ10:Y12, the lower OPV performance, when comparing 2MeTHF and CPME, is attributed to the oversized crystallites and the random orientation as measured by GIWAXS.

In summary, we propose the use of next-generation bioderivable solvents for the development of high-performance organic solar cells. Through extensive optoelectronic and morphological comparison with a petroleum-based solvent, 1,2-xylene, we highlight that there is no compromise in the optoelectronic properties of the donor:acceptor blends. In particular, we tested bioderived 2MeTHF and CPME solvents for two scalable donors, FO6-T and PTQ10, blended with Y12. For both systems, 2MeTHF was the better solvent, delivering a PCE of 14.5% for the PTQ10:Y12 OPV and 11.4% for the FO6-T:Y12 OPV, including when the active layer was deposited by a doctor blade in air. The CPME-based OPVs showed a lower overall performance, which was mainly attributed to the random crystal orientation in films, as observed via GIWAXS. A voltage loss analysis of the best-performing OPVs showed that PTQ10:Y12-based devices have a low nonradiative voltage loss of about 0.21 V, on par with the best-performing polymer:NFA devices processed from chlorinated solvents, indicating the presence of donor:acceptor interfaces with low CT-state energetic disorder. Lastly, through light-dependent OPV characterization, we showed that both systems have bimolecular recombination, with PTQ10:Y12 showing a light-independent FF. Overall, this work demonstrates high-performing OPVs processed from next-generation biorenewable solvents without the use of toxic additives with efficiencies comparable to those processed from petroleum-based and toxic solvents.

## Experimental Procedures

### Materials

PTQ10 and BTP-4F-12 (Y12) were purchased from Brilliant Matters, with a molecular weight of 46 kDa, as measured by the supplier. FO6-T was synthesized with a MW of 205 kDa. Molecular weight analysis was carried out on an analytical GPC Agilent Technologies 1200 series GPC equipped with a RI and UV detector running in chlorobenzene at 80 °C. Two PL mixed-B columns were set up, and narrow polydispersity standards were used to calibrate the system.

### Solvents

The biorenewable 2MeTHF (anhydrous, >99%) with and without BHT stabilizers was purchased from Merck. Similar results were obtained for both solvents; here we show results from 2MeTHF containing 250 ppm BHT as a stabilizer. All solvents used for device fabrication and thin film characterization were anhydrous and were purchased from Merck.

### Device Fabrication and Characterization

Organic solar cells were developed in an inverted device structure on prepatterned indium tin oxide (ITO) on glass. Prepatterned ITO (180 nm thick with 7–9 Ω resistance) substrates were cleaned in sequential sonication rounds of distilled water, followed by acetone and isopropanol, and 7 min of oxygen plasma treatment. As an electron-transporting layer, ZnO was spin-coated at 4000 rpm for 40 s from a 219.5 mg zinc acetate solution in a mixture of 60.4 μL of ethanolamine and 2 mL of 2-methoxyethanol. The film was annealed at 180 °C for 15 min, prior to the active layer deposition. PTQ10 and FO6-T were blended with Y12 in a 1:1.2 ratio, forming total concentrations of 18 and 22 mg/mL. Solutions were heated at 55 °C for at least 15 min prior to the deposition. For the spin-coated devices, ITO substrates were transferred in a nitrogen-filled glovebox for the active layer deposition. PTQ10:Y12 blend was spin-coated at 2500 rpm for 45 s, and FO6-T:Y12 at 3000 rpm for 45 s followed by thermal annealing at 100 °C for 10 min. For the doctor-bladed devices, the substrates were heated at a temperature of 40 °C. An active layer volume of 20 μL was dropped onto the substrates, and then the PTQ10:Y12 films were coated with a blade speed of 30 mm/s, while the FO6-T:Y12 films were coated at 15 mm/s. The same annealing and evaporation procedures as for the spin-coated devices were applied to the doctor-blade devices. 10 nm of MoOx used as the hole transporting layer and 100 nm of Ag for the top contact were thermally evaporated through shadow masks under a high vacuum (10^–6^ mbar), resulting in a pixel area of 0.045 cm^2^. The active layer of the spin-coated devices was conducted in a nitrogen-filled glovebox, where the doctor blade was processed in the air. The thickness of the doctor-bladed films and spin-coated films is in the same range of ∼100 nm.

### OPV Characterization

Current–voltage measurements were recorded with a 4200 Keithley Source–Measure unit with the use of an Oriel Instruments Solar Simulator with a xenon lamp, calibrated with a Newport silicon cell to provide AM1.5 G. OPV testing was conducted in air. For the low-light-intensity measurements ThorLabs 2 × 2 in. absorptive ND filters were used, with the optical density varying from 0.1 to 3. External quantum efficiency (EQE) was measured with a Quantum Design PV300 system in air. OPV stability was determined in the inverted devices at maximum power point tracking (MPP) under a nitrogen flow.

### Air Photoemission Spectroscopy (APS) and Kelvin Probe (KP)

The work function and the HOMO level of the organic semiconducting films were measured with a KP Technology SKP5050 Scanning Kelvin Probe and an APS02 Air Photoemission System, respectively. The thin films were spin-cast onto the ITO substrates. The contact potential difference was estimated relative to a reference sample (freshly polished silver) with work function 4.7 eV using APS.

### GIWAXS and GISAXS

GIWAXS measurements were performed using a Xeuss 2.0 SAXS/WAXS laboratory beamline with a Cu X-ray source (8.05 keV, 1.54 Å) and a Pilatus3R 300 K detector. The incidence angle was 0.2°. Thin films were prepared on silicon substrates.

### Optical and Electrical Characterization

UV–vis measurements were conducted on spin-coated organic semiconducting thin films on glass substrates by using a UV-1601 Shimadzu spectrometer.

### EL and PL

Photoluminescence and electroluminescence measurements were carried out by using an Andor iDUS InGaAs detector, and a laser with a wavelength of 485 nm was used as the excitation source for photoluminescence. Highly sensitive EQE measurements were carried out using a home-built system with a lock-in amplifier.

## Supporting Information Available

Solubility limit of polymers and NFA, contact angle measurements, UV–vis and PL on thin films, normal architecture OPVs, stability of OPVs, SCLC mobility analysis, electroluminescence, radiative voltage losses analysis, light intensity dependence characterization of OPVs, statistics of doctor-bladed OPVs, and GIWAXS and GISAXS analyses (PDF)
