# Supplementary material for: Biorenewable Solvents for High-Performance Organic Solar Cells
Source: ACS Energy Lett. 2023 Jun 16;8(7):3038–47. doi: 10.1021/acsenergylett.3c00891 (PMC10353010; doi:10.1021/acsenergylett.3c00891)
Supplement: Supplementary file 1 — nz3c00891_si_001.pdf [file nz3c00891_si_001.pdf]

Supporting Information

Biorenewable Solvents For High Performing Organic Solar Cells

Julianna Panidi[⊥,], Eva Mazzolini[⊥,§], Flurin Eisner[‖], Yuang Fu[╧], Francesco Furlan[⊥], Zhuoran Qiao[⊥], Martina Rimmele[⊥], Zhe Li[§], Xinhui Lu[╧], Jenny Nelson[‖], James R. Durrant[⊥,⫫], Martin Heeney[⊥,○], Nicola Gasparini[⊥]*

[⊥] Department of Chemistry & Centre for Processable Electronics, Imperial College London, London, W12 0BZ, UK

[§] School of Engineering and Materials Science (SEMS), Queen Mary University of London, London E1 4NS, UK

[‖] Department of Physics & Centre for Processable Electronics, Imperial College London, London, W12 0BZ, UK

[╧] Department of Physics, The Chinese University of Hong Kong, Shatin, Hong Kong SAR 999077, P. R. China

[○] King Abdullah University of Science and Technology (KAUST), KAUST Solar Center (KSC), Physical Sciences and Engineering Division (PSE), Thuwal, 23955–6900 Saudi Arabia

[⫫] Department of Materials Science and Engineering and SPECIFIC IKC, Swansea University, Bay Campus, Fabian Way, Swansea, Wales SA1 8EN, UK

Corresponding author email: j.panidi@imperial.ac.uk

Figure S1: Picture of PM6 in the solvents CPME (left) and 2MeTHF (right). A lot of particles are still suspended at a concentration of 0.05 mg/ml......5
Figure S2: UV-Vis absorption measurements at different concentrations and the corresponding calibration curves for FO6-T in 1,2-xylene, CPME and 2MeTHF......6
Figure S3: UV-Vis absorption measurements at different concentrations and the corresponding calibration curves for PTQ10 in 1,2-xylene, CPME and 2MeTHF. 7
Figure S4: UV-Vis absorption measurements at different concentrations and the corresponding calibration curves for Y12 in 1,2-xylene, CPME and 2MeTHF......8
Figure S5: Solubility limit as extracted from UV-Vis characterisation for FO6-T, PTQ10 and Y12 in three tested solvents. 9
Figure S6: Contact angle measurements for FO6-T, PTQ10 and Y12 deposited from the three tested solvents: 2MeTHF, CPME and xylene. Droplets of water Diiodo-methane (DI), ethylene glycol (EG) and water (DI) were dropped on the thin film surface and to calculate the contact angle. The surface energy values were calculated according to Owens, Wendt, Rable and Kaekble (OWRK) method. The contact angle goniometer was purchased from Ossila......10
Figure S7: UV-Vis and PL spectra for a) Y12, b) PTQ10 and c) FO6-T thin films in 1,2-xylene, 2MeTHF and CPME. 12
Figure S8: UV-Vis and PL for a) PTQ10:Y12, b) PTQ10:Y12 of thin films in 1,2-xylene, 2MeTHF and CPME......13
Figure S9: Normal architecture OPVs developed from FO6-T:Y12 and PTQ10:Y12 in 2MeTHF......14
Figure S10: EQE and integrated Jsc from a) PTQ10:Y12 and b) FO6-T:Y12 OPVs processed from 2MeTHF, xylene and CPME. 15
Figure S11: Maximum power point tracking for FO6-T:Y12 and PTQ10:Y12 OPVs processed from 2MeTHF, upon nitrogen purging under 1 Sun conditions......16
Figure S12: LED light spectrum used for the stability characterisation......16
Figure S13: Hole carrier mobility plot for a) pristine FO6-T, b) pristine PTQ10, c) FO6-T:Y12, d) PTQ10:Y12 in 2MeTHF, CPME and 1,2-xylene. 18
Figure S14: Electron carrier mobility plot for a) pristine Y12, b) FO6-T:Y12, c) PTQ10:Y12 in 2MeTHF, CPME and 1,2-xylene......19
Figure S15: El for the PTQ10:Y12 and FO6-T:Y12 blends in 2MeTHF and CPME. 20
Figure S16: EQE for a) PTQ10:Y12 and b) FO6-T:Y12 in 2MeTHF......21
Figure S17: a) J_{sc} dependence of light intensity for FO6-T:Y12 with slope of 0.9 and PTQ10:Y12 with slope of 0.88 and b) J_{ph} variation on effective voltage. 22
Figure S18: Photogenerated current vs effective voltage for FO6-T:Y12 and PTQ10:Y12 OPVs, where the active layer was processed form a) 2MeTHF and b) 1,2-Xylene. 23
Figure S19: FO6-T:Y12 and PTQ10:Y12 OPVs processed from 2MeTHF via doctor blade a) J-V and b) EQE representative characteristics with the integrated short circuit current. 24
Figure S20: 2D GIWAXS plots of thin film as processed from a) 2MeTHF and b) CPME as well as (c) their linecuts extracted along in-plane (solid line) and out-of-plane (dashed line) directions. 25
Figure S21: The 2D GISAXS plots of 2MeTHF-processed (a) FO6-T:Y12, (b) PTQ10:Y12 blend films. The corresponding plots for CPME-processed films are shown in (c)-(d). A summary of in-plane line cuts extracted at the Yoneda peak is shown in (e). 25

Table S1: Summary table of non-halogenated and non-aromatic solvents used for OPV fabrication.

Solvent	Material	PCE	Reference
Terpinolene	PTB7:PC71BM	6.42 %	[1]
Eucalyptol	PBDTTPD:PCBM	2.40 %	[2]
Limonene: 2MeTHF	FTAZ:IT-M	12.2 %	[3]
Limonene	PTzBI-Si:PNDICl	4.20 %	[4]
Water:Ethanol	PFO3/PFO4:PCBO-12	3.03 %	[5]
Ethanol	PPDT2FBT-A:Bis-C60-A	0.75 %	[6]
Water:Ethanol	PPDT2FBT-A:P(NDIDEG-T	2.15 %	[7]
2MeTHF	PTzBI-Si:N2200	10.10 %	[8]
2MeTHF: DBE	PTzBi-OF:PS1	13.75 %	[9]
2MeTHF*	PTQ10:Y12	14.50 %	This work
2MeTHF*	FO6-T:Y12	11.40 %	This work
CPME	PTQ10:Y12	4.90 %	This work
CPME	FO6-T:Y12	3.30 %	This work

*biosourced 2MeTHF

Table S2: Direct comparison of the safety 1,2-xylene and 2MeTHF solvents from the SDS forms provided by the supplier (Merck).

	Info	1,2-xylene	2-MeTHF
	Production	Petroleum-based	Biosourced
		Non-halogenated, aromatic	Non-halogenated & non-aromatic
a	Workplace control parameters (TWA: Time-weighted average: a measurement of average exposure limit over a certain period)	50 ppm, according to Europe. Commission Directive 2000/39/EC & UK. EH40 WEL - Workplace Exposure Limits	Contains no substances with occupational exposure limit values.
	Acute toxicity, median lethal dose (LD50, oral)	3,523 mg/kg	300 - 2,000 mg/kg
	Toxicity to fish	2.6 mg/ml	100 mg/ml
	Flammable	3	2
	Eye damage/irritation	2	1
	Detailed Health Phrases (H)		
		May be fatal if swallowed and enters the airways. (H304)	Harmful if swallowed. (H302)
		May cause respiratory irritation. (H335)	Causes serious eye damage. (H318)
		Harmful to aquatic life with long-lasting effects. (H412)	Causes skin irritation. (H315)
		Harmful in contact with skin or if inhaled. (H312 + H332)	

Solubility Limit of Polymers and NFA

Solubility limit measurements as performed for the 2 polymers PTQ10 and FO6-T, and the NFA Y12 in their respective solvents, including 2MeTHF, CPME and xylene at room temperature. The limit of the solubility was determined *via* a standard calibration curve method[10]. UV-Vis absorption measurements of polymer solutions in the three solvents were performed at different concentrations (0.005 mg/mL to 0.05 mg/mL) and the calibration curve constructed. Saturated solutions of the polymers were prepared in 1,2-xylene, CPME and 2MeTHF continually adding material to the solution, and subsequent centrifuging of the solutions (20 min, 10000 rpm), an aliquot of the solution was used to prepare a diluted sample for UV-Vis absorption measurement. The concentration of the saturated solution was then determined by using the linear equation of the standard calibration curve.

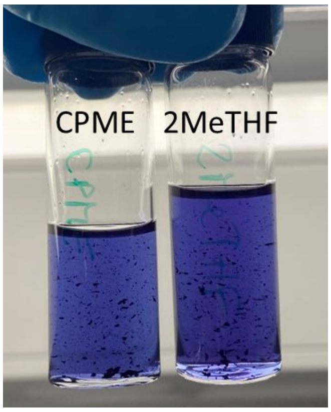

Figure S1: Picture of PM6 in the solvents CPME (left) and 2MeTHF (right). A lot of particles are still suspended at a concentration of 0.05 mg/ml.

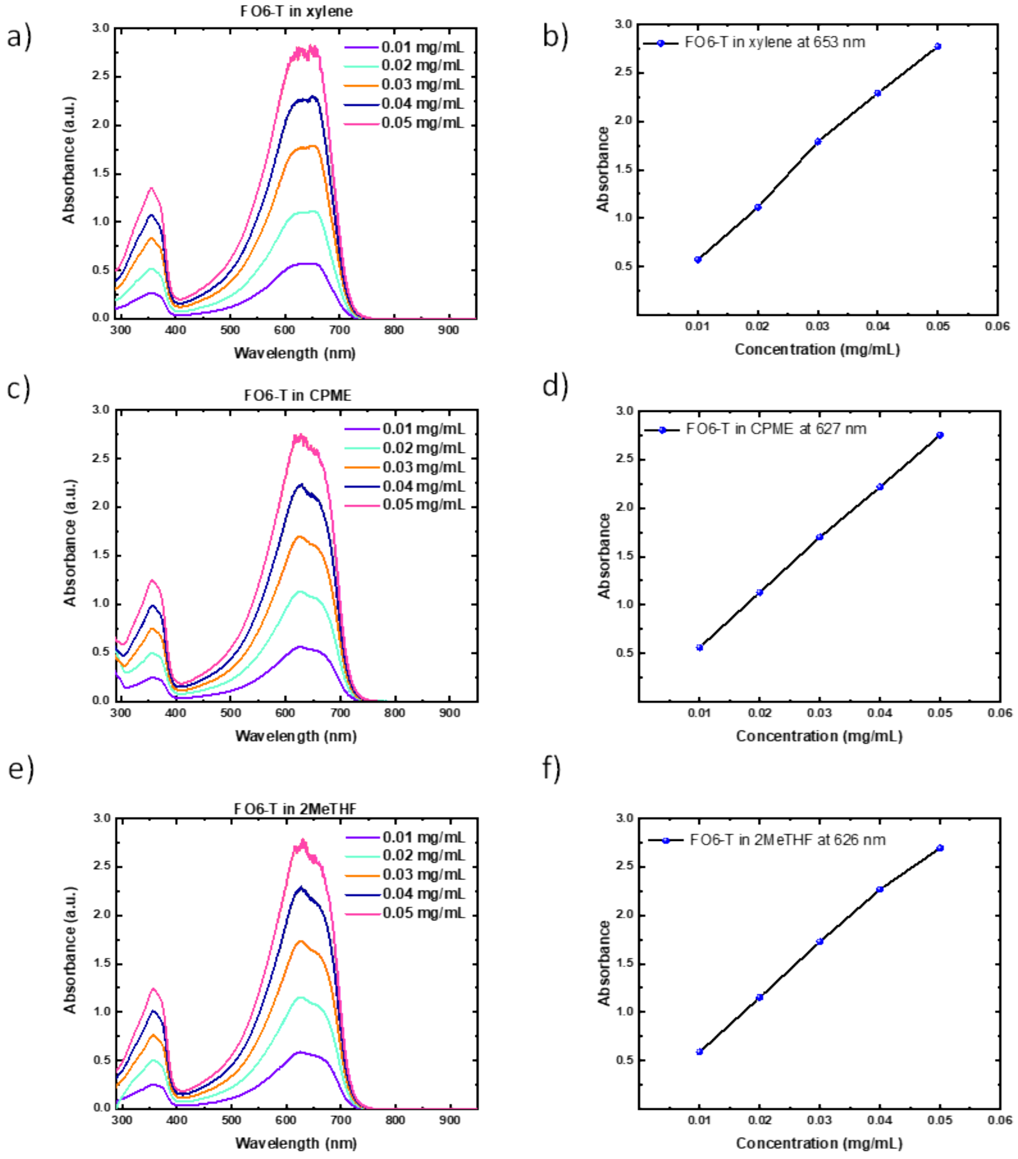

Figure S2: UV-Vis absorption measurements at different concentrations and the corresponding calibration curves for FO6-T in 1,2-xylene, CPME and 2MeTHF.

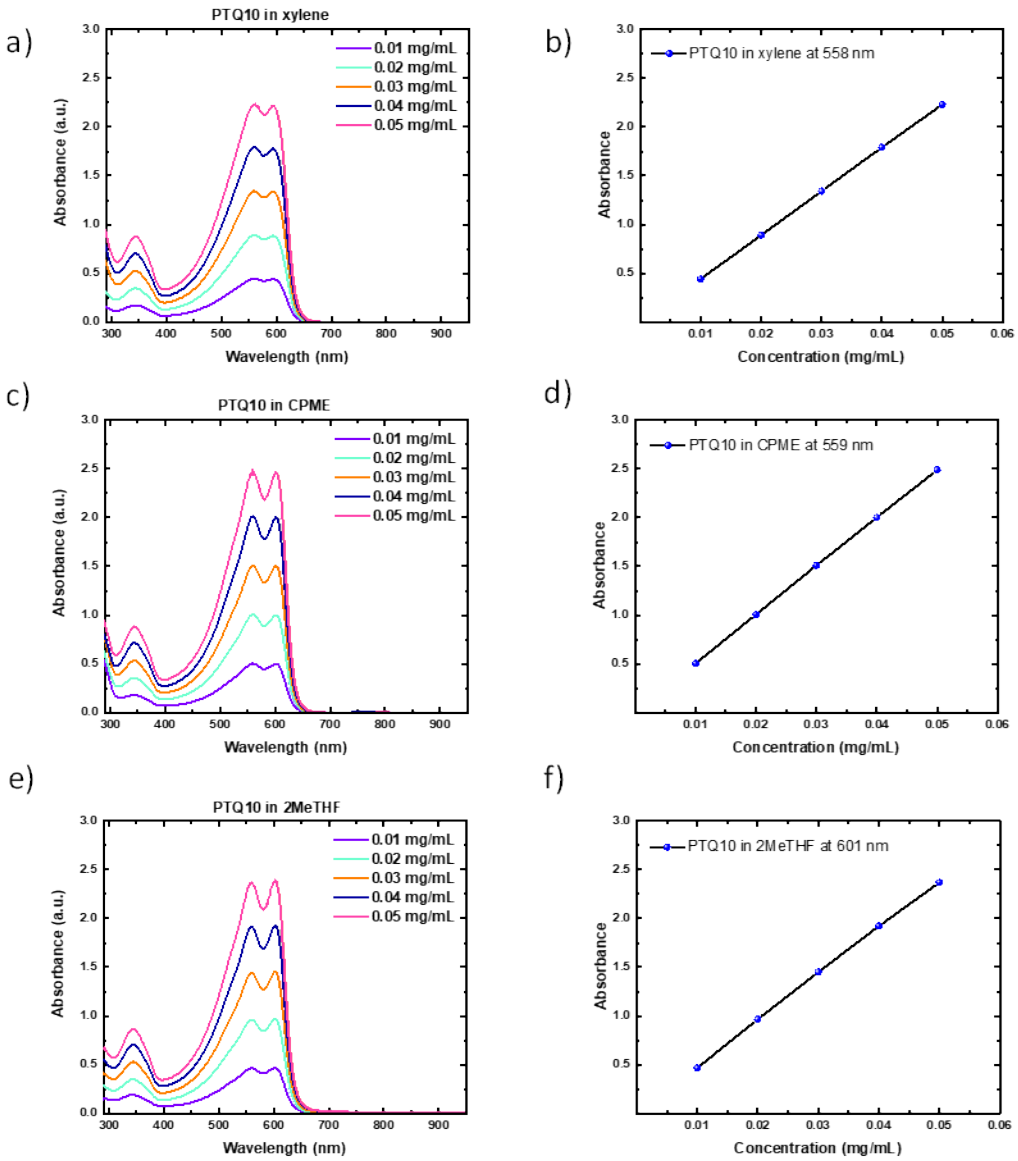

Figure S3: UV-Vis absorption measurements at different concentrations and the corresponding calibration curves for PTQ10 in 1,2-xylene, CPME and 2MeTHF.

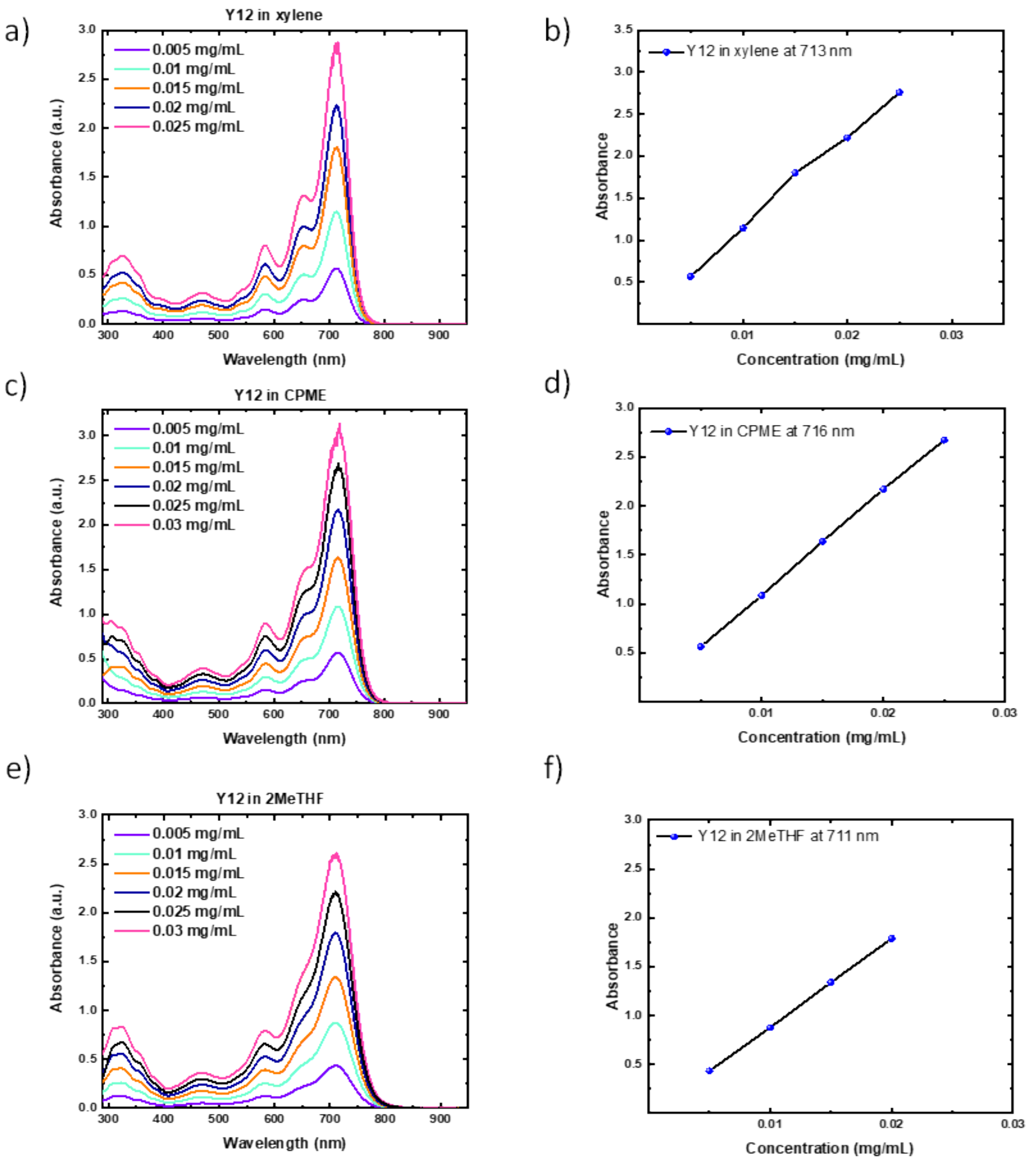

Figure S4: UV-Vis absorption measurements at different concentrations and the corresponding calibration curves for Y12 in 1,2-xylene, CPME and 2MeTHF.

Table S3: Data summary as extracted from Figures S1-S3 which they were then used to calculate the solubility limit as displayed in Figure S4.

		**Abs at λ_{max}**	**Slope**	**Intersect**
FO6-T	1,2-xylene	1.23	55.81	0.03
	CPME	1.25	54.76	0.03
	2MeTHF	1.17	53.28	0.09
PTQ10	1,2-xylene	1.09	44.82	-0.01
	CPME	0.52	49.70	0.01
	2MeTHF	1.47	47.64	0.01
Y12	1,2-xylene	1.53	109.21	0.06
	CPME	1.05	106.02	0.04
	2MeTHF	0.99	90.52	-0.02

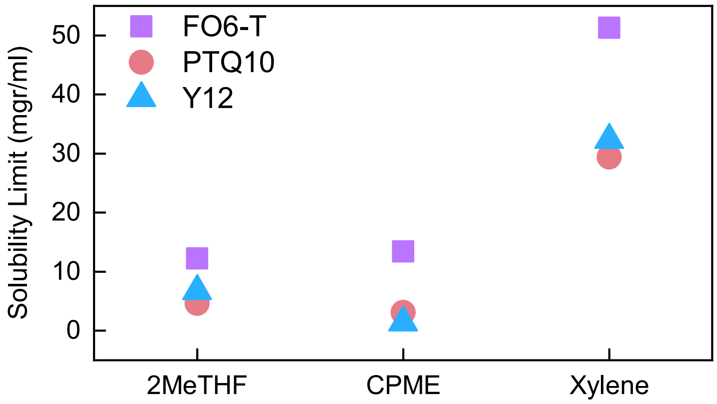

Figure S5: Solubility limit as extracted from UV-Vis characterisation for FO6-T, PTQ10 and Y12 in three tested solvents.

Contact Angle Measurements

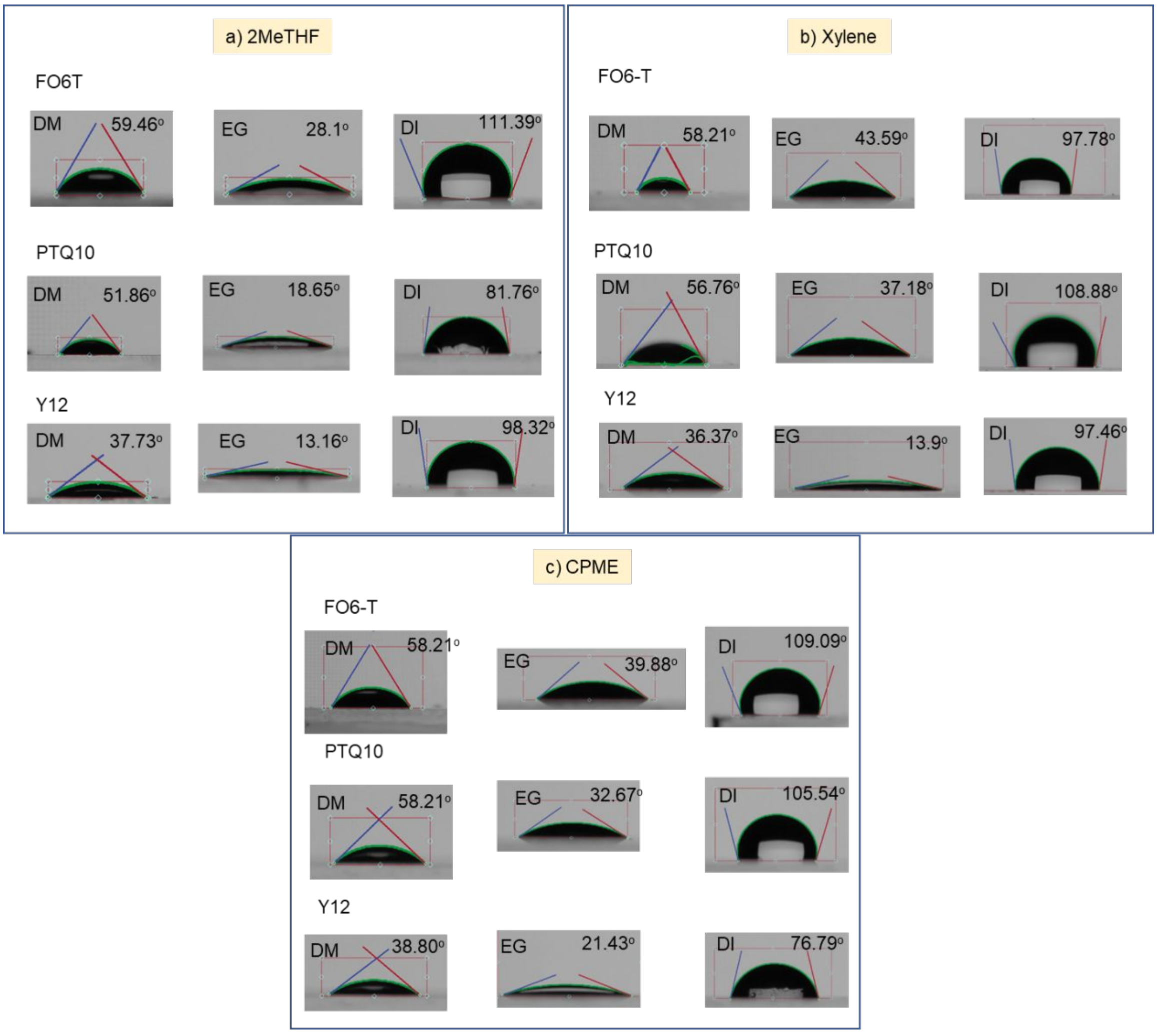

Figure S6: Contact angle measurements for FO6-T, PTQ10 and Y12 deposited from the three tested solvents: 2MeTHF, CPME and xylene. Droplets of water Diiodo-methane (DI), ethylene glycol (EG) and water (DI) were dropped on the thin film surface and to calculate the contact angle. The surface energy values were calculated according to Owens, Wendt, Rable and Kaekble (OWRK) method. The contact angle goniometer was purchased from Ossila.

Obtaining the surface energy values allowed us to calculate the intermixing parameter (χ) according to Flory Huggins theory; $\chi \propto (\sqrt{\gamma_1} - \sqrt{\gamma_2})^2$, where 1 and 2 are the individual components in blends. As shown in Table S4 higher value for χ is obtained for both systems when preparing the films from xylene, suggesting that less interaction is occurring between the two components in xylene compared with 2MeTHF and CPME. The analysis was based on previously published studies[11].

Table S4: Surface energy and Flory-Huggin's interaction parameter χ as calculated from the surface energy of the individual components

**Solvent**	**Material**	**Surface Free Energy (mN/m)**	χ
2MeTHF	FO6-T	40.4	0.55
	PTQ10	42.5	0.33
	Y12	50.4	-
CPME	FO6-T	38.75	0.43
	PTQ10	39.15	0.39
	Y12	47.44	-
Xylene	FO6-T	36.15	1.2
	PTQ10	40.01	0.64
	Y12	50.8	-

UV-Vis and PL

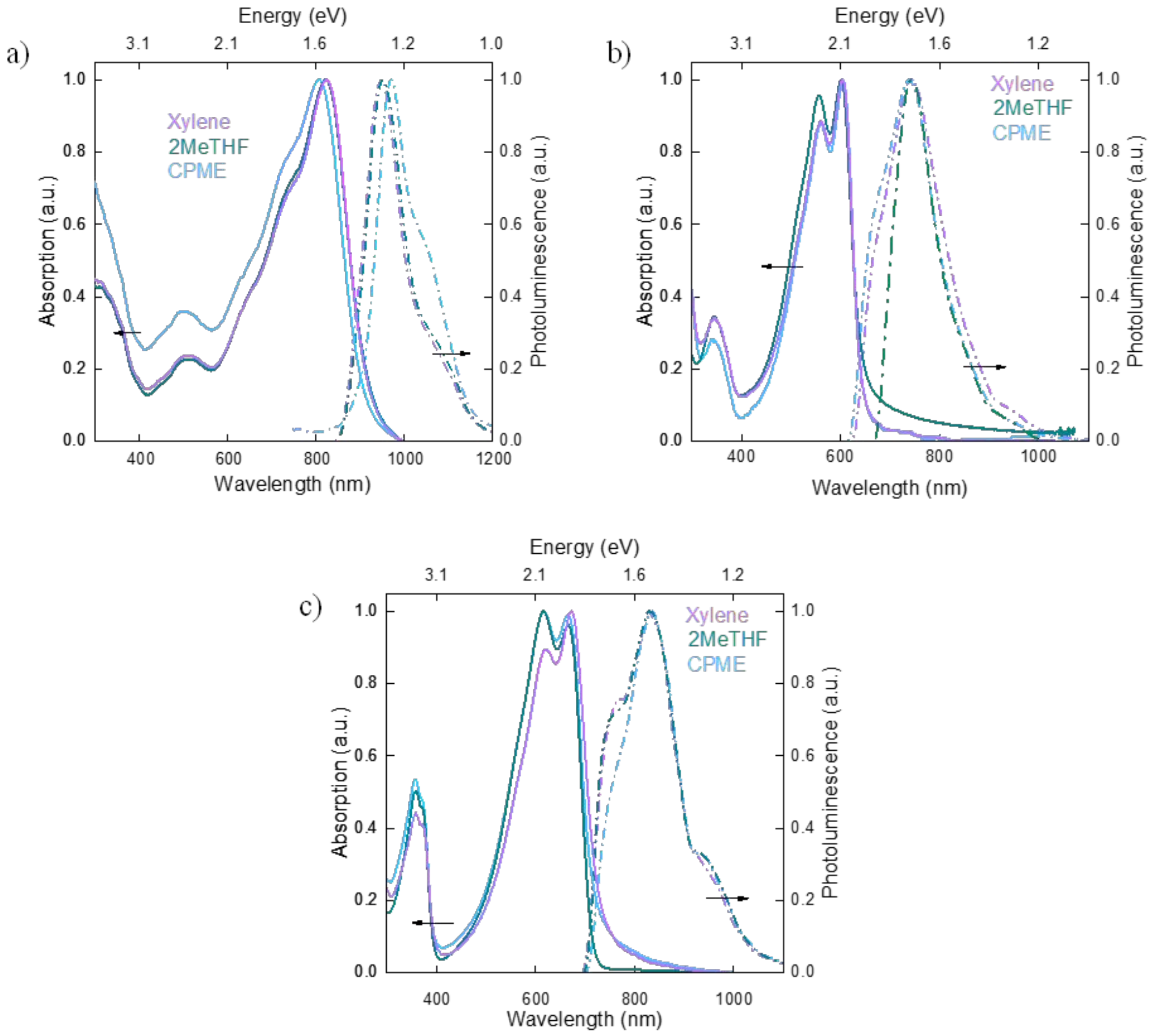

Figure S7: UV-Vis and PL spectra for a) Y12, b) PTQ10 and c) FO6-T thin films in 1,2-xylene, 2MeTHF and CPME.

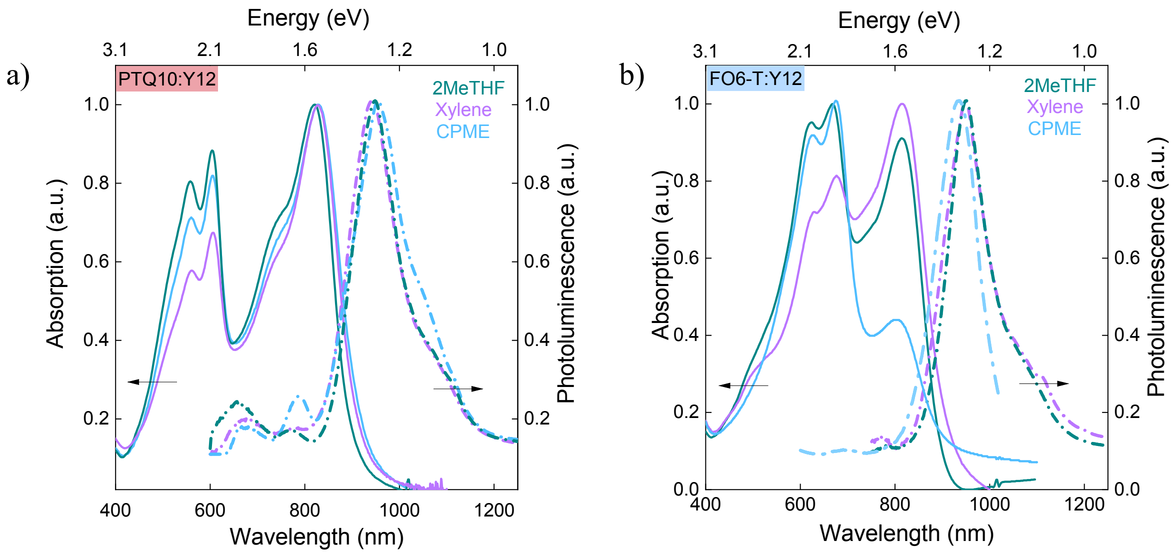

Figure S8: UV-Vis and PL for a) PTQ10:Y12, b) PTQ10:Y12 of thin films in 1,2-xylene, 2MeTHF and CPME.

Normal architecture OPVs

Device structure consists of ITO/PEDOT:PSS/AL/ETL/Ag. For the PTQ10:Y12 OPVs PDINO was used as ETL and for the FO6-T:Y12 DPO. PEDOT:PSS was purchased from Clevios and was filtered with 0.45 µm PTFE filter prior spin-coating. PEDOT:PSS solution was spin-coated at 4000 rpm for 40 seconds, followed by thermal annealing at 150°C. Donor:NFA blends were prepared and deposited as described in the OPV Fabrication section. For the ETLs PDINO resulted in the highest performing PTQ10:Y12 OPVs, whereas for the FO6-T:Y12 OPVs DPO worked better. 0.5 mg/ml of PDINO were dissolved in methanol and 0.5 mg/ml of DPO in isopropanol. ETLs were spin coated at 2000 rpm for 30 seconds. For the top electrode, 100 nm of Ag were deposited via thermal evaporation. Figure S9 shows the J-V characteristics of the normal architecture OPVs and their parameters are presented in Table S6.

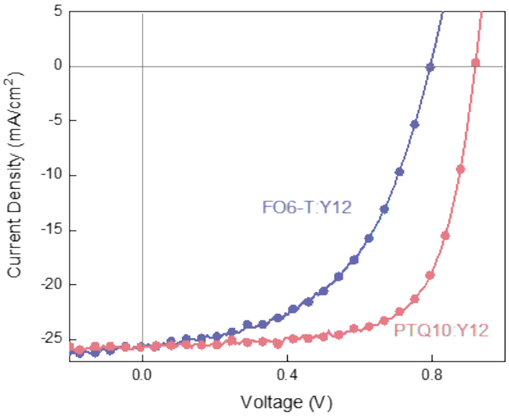

Figure S9: Normal architecture OPVs developed from FO6-T:Y12 and PTQ10:Y12 in 2MeTHF.

Table S5: Normal architecture OPV parameters for PTQ10:Y12 and FO6-T:Y12 devices when active layer was processed from 2MeTHF showing the mean, std deviation and maximum value obtained in brackets from 15 devices in normal architecture.

	J_{sc} (mA/cm^2)	V_{oc} (V)	FF	PCE
**PTQ10:Y12**	22.36 ± 2.33 (25.2)	0.85 ± 0.005 (0.86)	0.62 ± 0.03 (0.54)	12.18 ± 2.4 (14)
**FO6-T:Y12**	24.67 ± 2.47 (26.4)	0.76 ± 0.02 (0.79)	0.49 ± 0.03 (0.52)	9.62 ± 1.48 (10.8)

EQE with Integrated Jsc current of Inverted OPVs

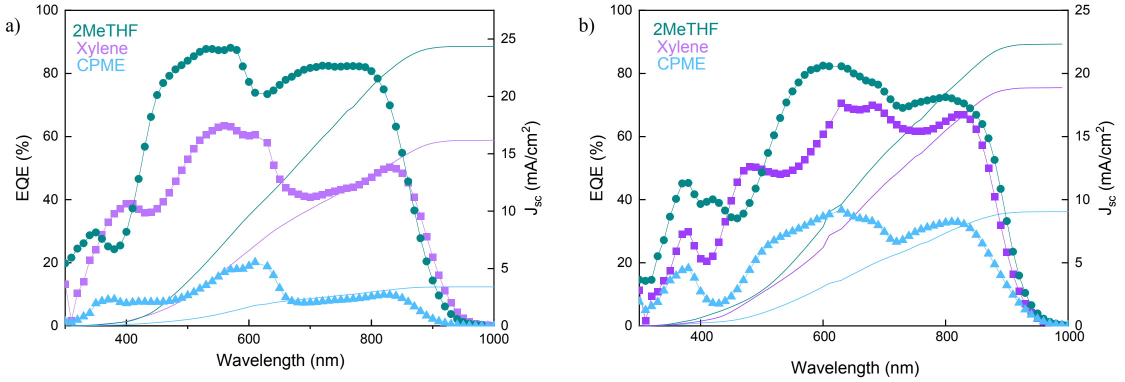

Figure S10: EQE and integrated Jsc from a) PTQ10:Y12 and b) FO6-T:Y12 OPVs processed from 2MeTHF, xylene and CPME.

Stability of inverted OPVs

The stability of the inverted structure 2MeTHF-based OPVs was evaluated under 1 Sun intensity using a series of LEDs with light spectrum as presented in Figure S12.

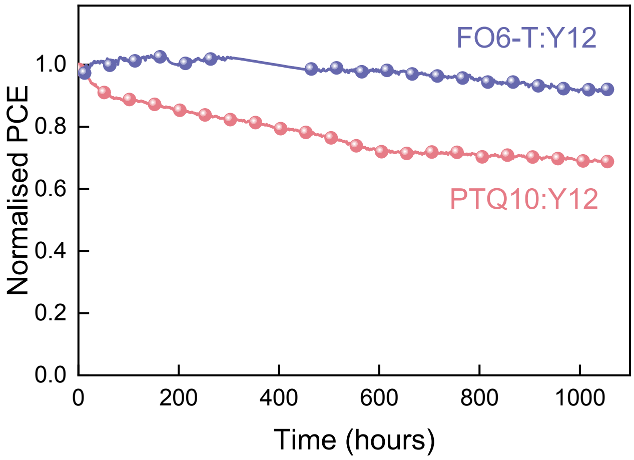

Figure S11: Maximum power point tracking for FO6-T:Y12 and PTQ10:Y12 OPVs processed from 2MeTHF, upon nitrogen purging under 1 Sun conditions.

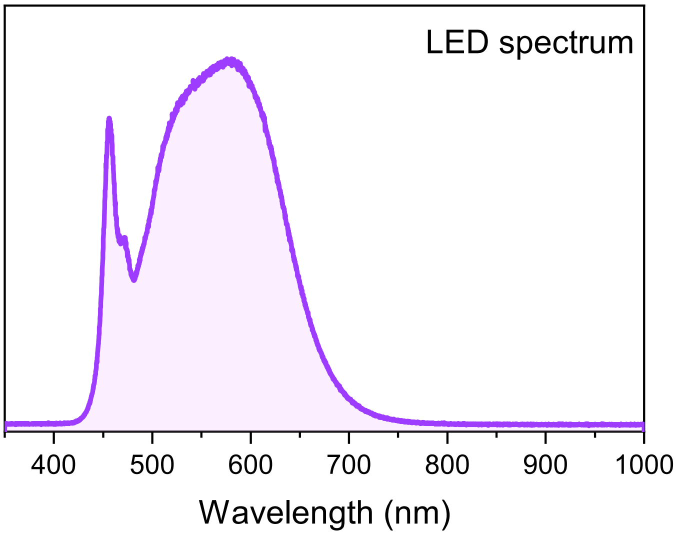

Figure S12: LED light spectrum used for the stability characterisation.

SCLC Mobility Analysis

Electron only and hole only devices were fabricated onto patterned ITO glass, previously cleaned following the same protocol used for solar cells. Hole only and electron only devices devices are fabricated with the structure ITO/PEDOT:PSS/Active Layer/Au and ITO/ZnO/PEIE/Active Layer/Ca/Al, respectively. PEDOT:PSS (Al4083) is spin coated to obtain a layer with a thickness of 40nm. The same solution preparation and deposition protocol was conducted for the ZnO as reported in the OPV fabrication. ZnO is further rinsed with PEIE diluted at 0.5% wt in 2-Methoxyethanol. Single components solutions were prepared in 2MeTHF, CPME and 1,2-xylene with a concentration of 10 mg/mL and spin coated on substrates at 2000 rpm for 45s. All blends active layer were deposited with a similar procedure employed for OPV fabrication. The thickness of all the tested active layers was measured using Dektak profilometer and all J-V curves were recorded with a Keithley 2400.

To extrapolate mobility values the Murgatroyd equation was employed:

$$J = \frac{9}{8}\mu_0 \varepsilon \frac{V^2}{d^3} exp\left(0.89\gamma\sqrt{\frac{V}{d}}\right)$$

Where ε is the relative dielectric constant of the material (3 was assumed), ε_0 the vacuum permittivity, μ the mobility, *d* the film thickness, γ is the field activation factor of mobility, and *V* is the applied voltage.

When extracting the values of μ_0 and γ from the experimental data, the value of μ at any field *E* can be obtained by using the Poole–Frenkel expression:

$$\mu = \mu_0 exp(\gamma\sqrt{E})$$

All extracted mobility values are collected in Table 3 from the curves below (Figure S8 and S9):

Table S6: Electron and hole carrier mobilities as extracted from the devices.

		Electron mobility cm^2/Vs	Hole mobility cm^2/Vs
FO6-T:Y12	1,2-xylene	1.0×10^{-5}	2.8×10^{-4}
	CPME	4.5×10^{-7}	4.7×10^{-5}
	2MeTHF	1.8×10^{-5}	4.1×10^{-5}
PTQ10:Y12	1,2-xylene	6.4×10^{-6}	3.0×10^{-5}
	CPME	9.3×10^{-7}	2.4×10^{-5}
	2MeTHF	1.4×10^{-6}	5.5×10^{-5}

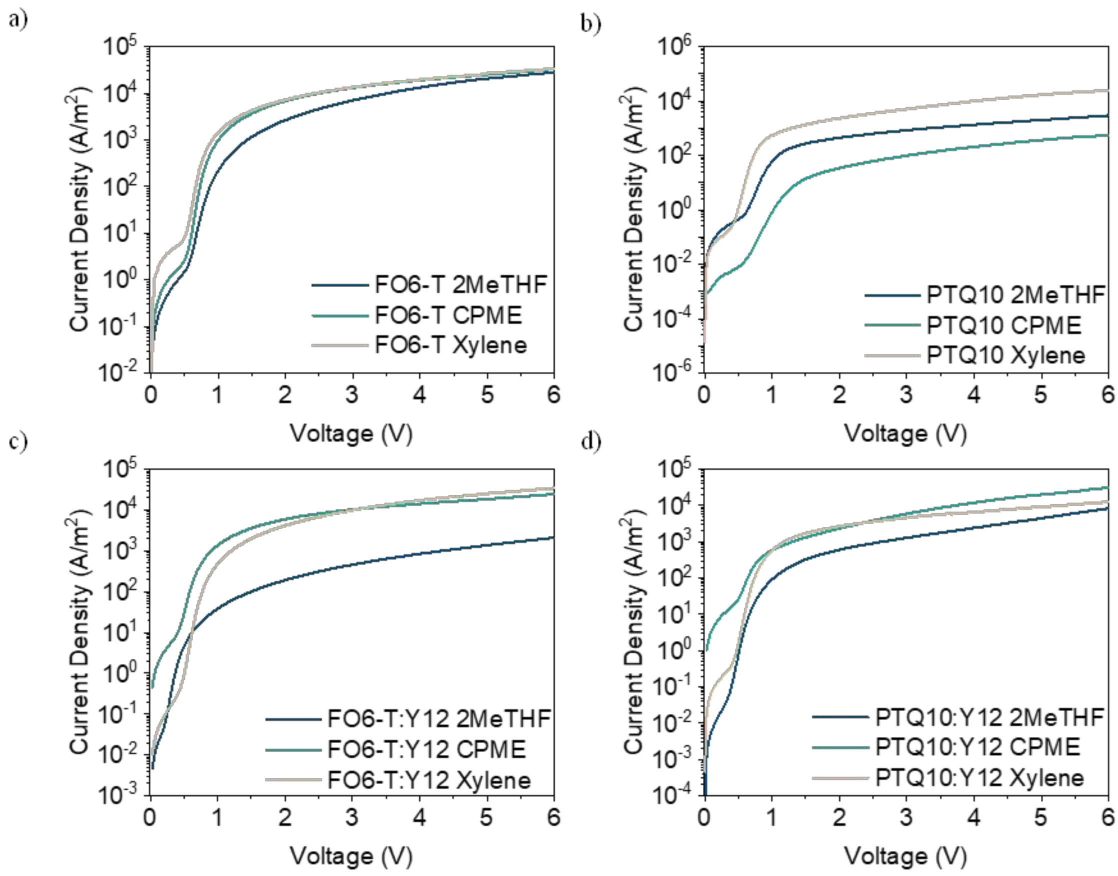

Figure S13: Hole carrier mobility plot for a) pristine FO6-T, b) pristine PTQ10, c) FO6-T:Y12, d) PTQ10:Y12 in 2MeTHF, CPME and 1,2-xylene.

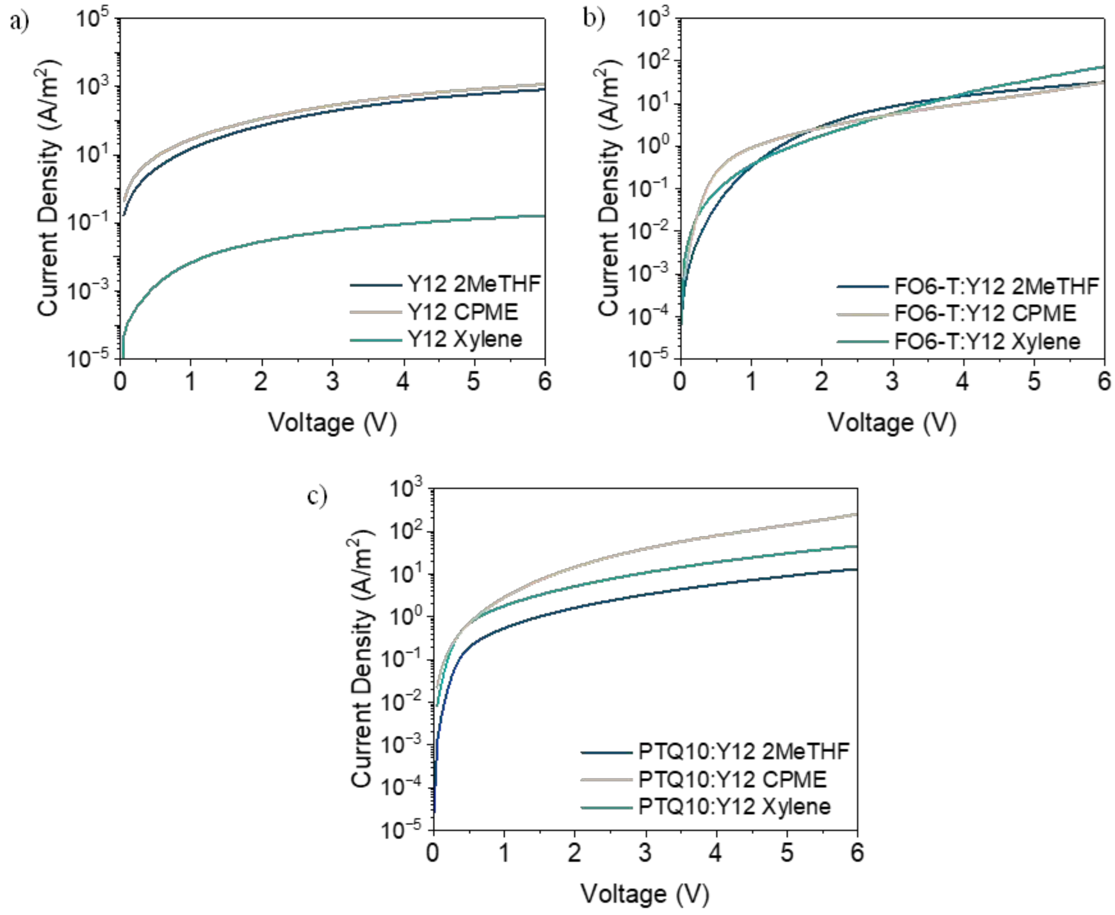

Figure S14: Electron carrier mobility plot for a) pristine Y12, b) FO6-T:Y12, c) PTQ10:Y12 in 2MeTHF, CPME and 1,2-xylene.

Electroluminescence

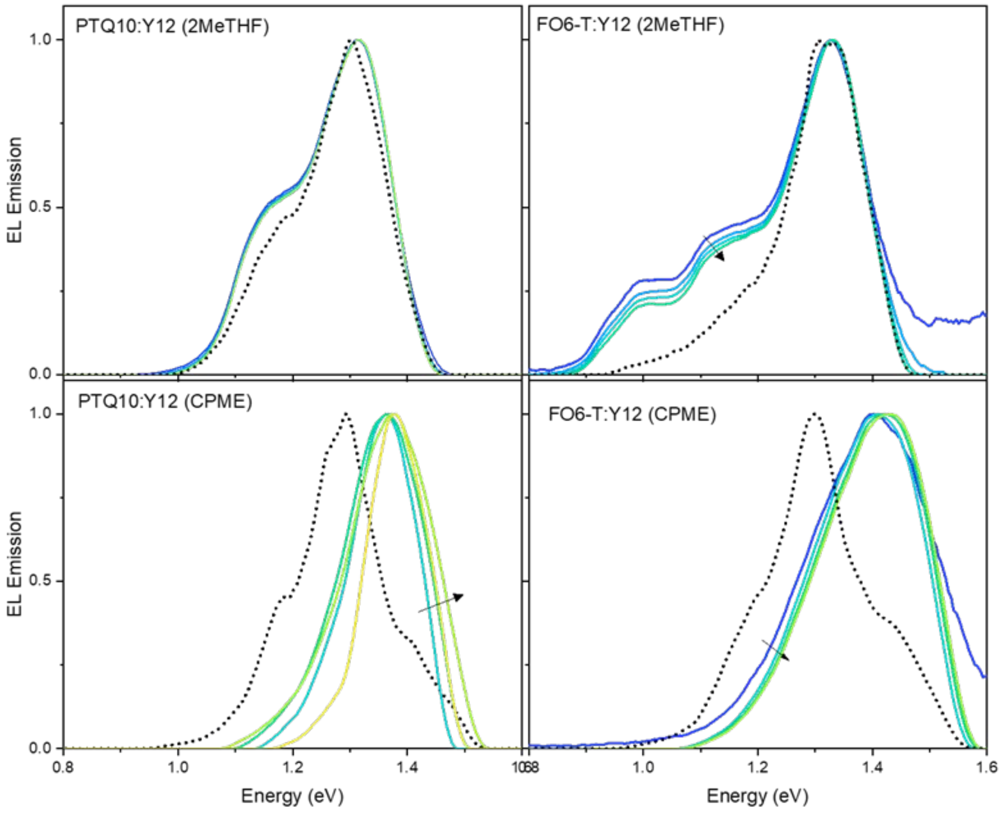

Figure S15: El for the PTQ10:Y12 and FO6-T:Y12 blends in 2MeTHF and CPME.

Radiative voltage losses analysis

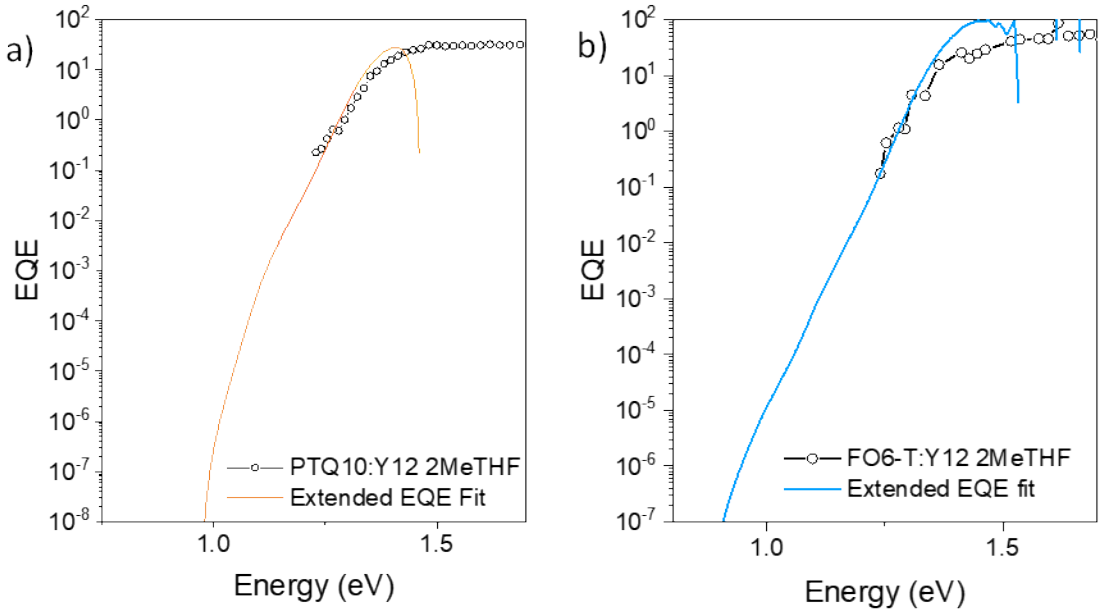

Figure S16: EQE for a) PTQ10:Y12 and b) FO6-T:Y12 in 2MeTHF

Table S7: Extracted radiative voltage losses from Figure S14.

	$V_{oc,rad}$	V_{oc}	$\Delta V_{oc,nrad}$
PTQ10:Y12	1.06	0.85	0.21
FO6-T:Y12	1.06	0.77	0.28

Light Intensity Dependence Characterisation of OPVs

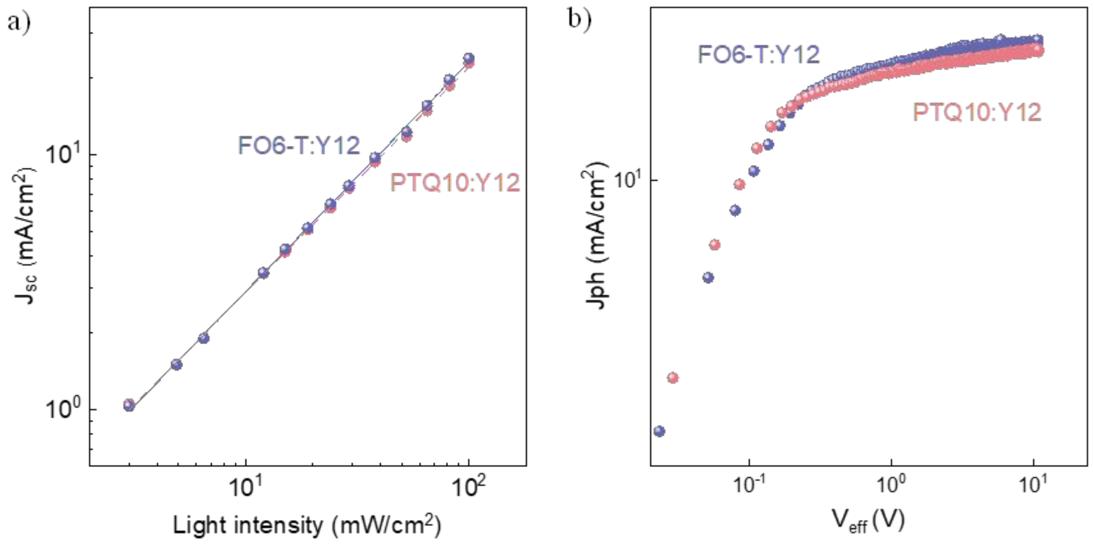

Figure S17: a) J_{sc} dependence of light intensity for FO6-T:Y12 with slope of 0.9 and PTQ10:Y12 with slope of 0.88 and b) J_{ph} variation on effective voltage.

The exciton generation rate was calculated based on the method reported before.[12] Photocurrent current density (J_{ph}) is defined by J_{ph}=J_l-J_d, where J_l is the current density under 100 mW/cm^2 and J_d under dark conditions. Effective voltage is defined as V_{eff}=V_0-V, where V_0 is the $J_{ph}(V_0)$=0, and V is the applied voltage. For the G_{max} calculation the J_{sat}=qG_{max}L, where q is the elementary charge and L is the thickness of the active layer. The thickness of PTQ10:Y12 thin film was found 110 nm and of the FO6-T:Y12 120 nm.

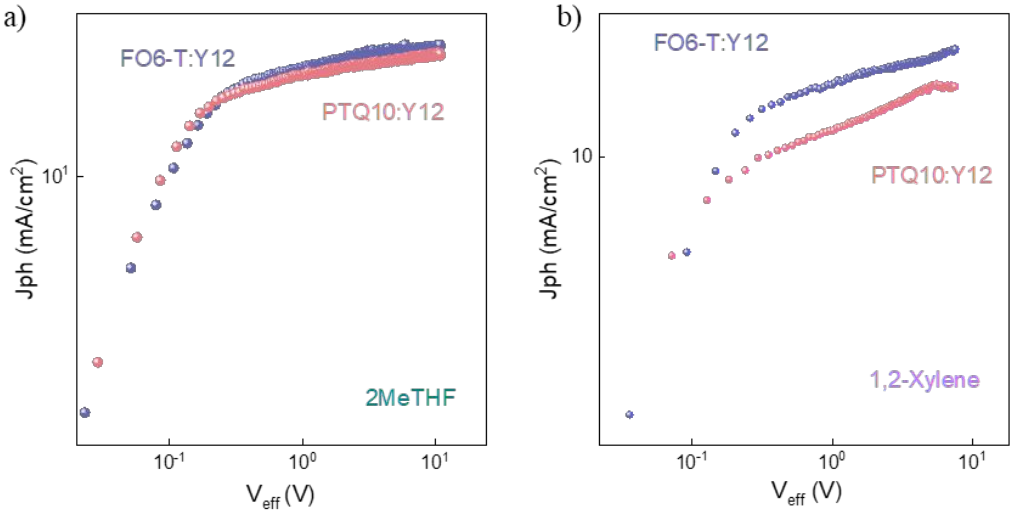

Figure S18: Photogenerated current vs effective voltage for FO6-T:Y12 and PTQ10:Y12 OPVs, where the active layer was processed form a) 2MeTHF and b) 1,2-Xylene.

Table S8: Calculated parameters as extracted from the light dependent J-V characterisation, demonstrating the saturation current (J_{SAT}) and the rate of free charge carrier generation in saturation, under maximum power point (G_{MPP})and under short circuit conditions (G_{SC}).

	J_{SAT} (mA cm $^{-2}$)	G_{MAX} ($s^{-1}cm^{-3}$)	G_{MPP} (%)	G_{SC} (%)
FO6-T:Y12				
Xylene	20.3	1.26 x 10^{22}	63.30	78.41
2MeTHF	27.8	1.73 x 10^{22}	69.02	86.33
PTQ10:Y12				
Xylene	15.76	9.85 x 10^{21}	55.13	74.36
2MeTHF	26.8	1.67 x 10^{22}	69.4	85.75

Statistics of Doctor blade OPVs

Table S9: OPV parameters doctor blade PTQ10:Y12 and FO6-T:Y12 devices when active layer was processed from 2MeTHF showing the mean, std deviation and maximum value obtained in brackets. Short circuit current value as extracted from the J-V and EQE measurements.

	Jsc (mA/cm^2)	Voc (V)	FF	PCE
		**PTQ10:Y12**		
2MeTHF	21.52 ± 1.33 (23.03)	0.851 ± 0.002 (0.855)	0.69 ± 0.01 (0.70)	12.64 ± 0.86 (13.8)
		**FO6-T:Y12**		
2MeTHF	22.94 ± 0.59 (23.79)	0.802 ± 0.008 (0.810)	0.57 ± 0.04 (0.63)	10.78 ± 1.17 (12)

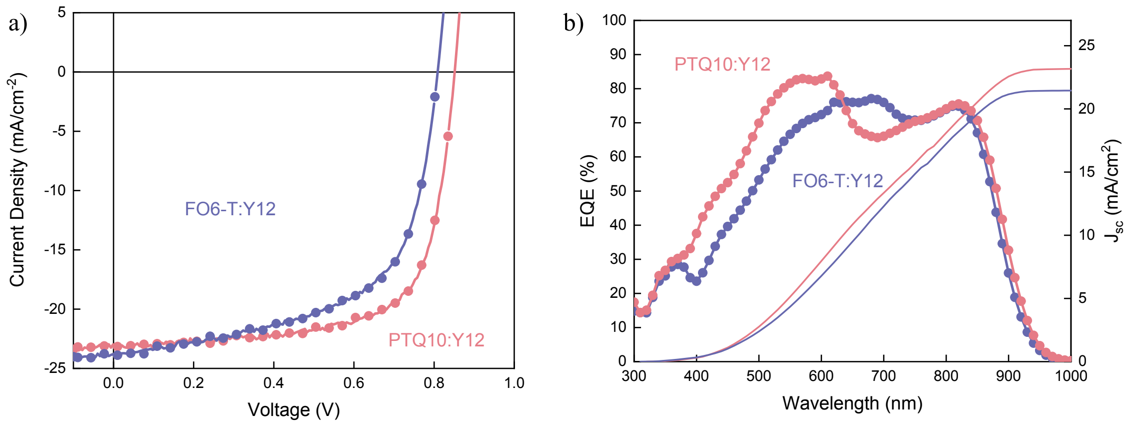

Figure S19: FO6-T:Y12 and PTQ10:Y12 OPVs processed from 2MeTHF via doctor blade a) J-V and b) EQE representative characteristics with the integrated short circuit current.

GIWAXS and GISAXS Characterisation

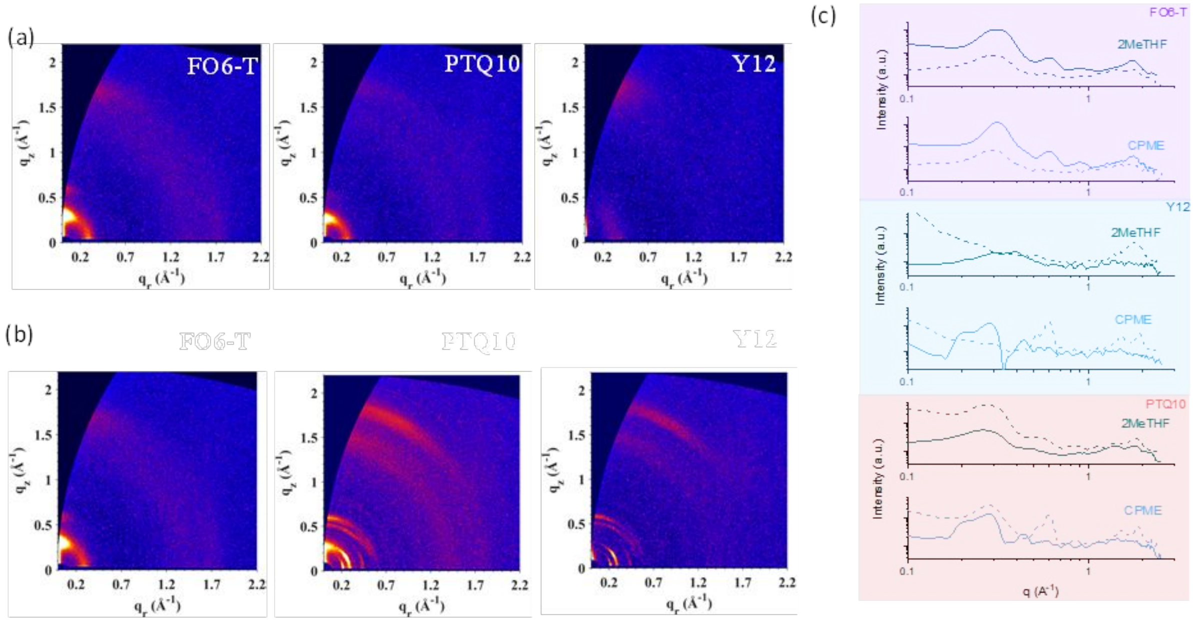

Figure S20: 2D GIWAXS plots of thin film as processed from a) 2MeTHF and b) CPME as well as (c) their linecuts extracted along in-plane (solid line) and out-of-plane (dashed line) directions.

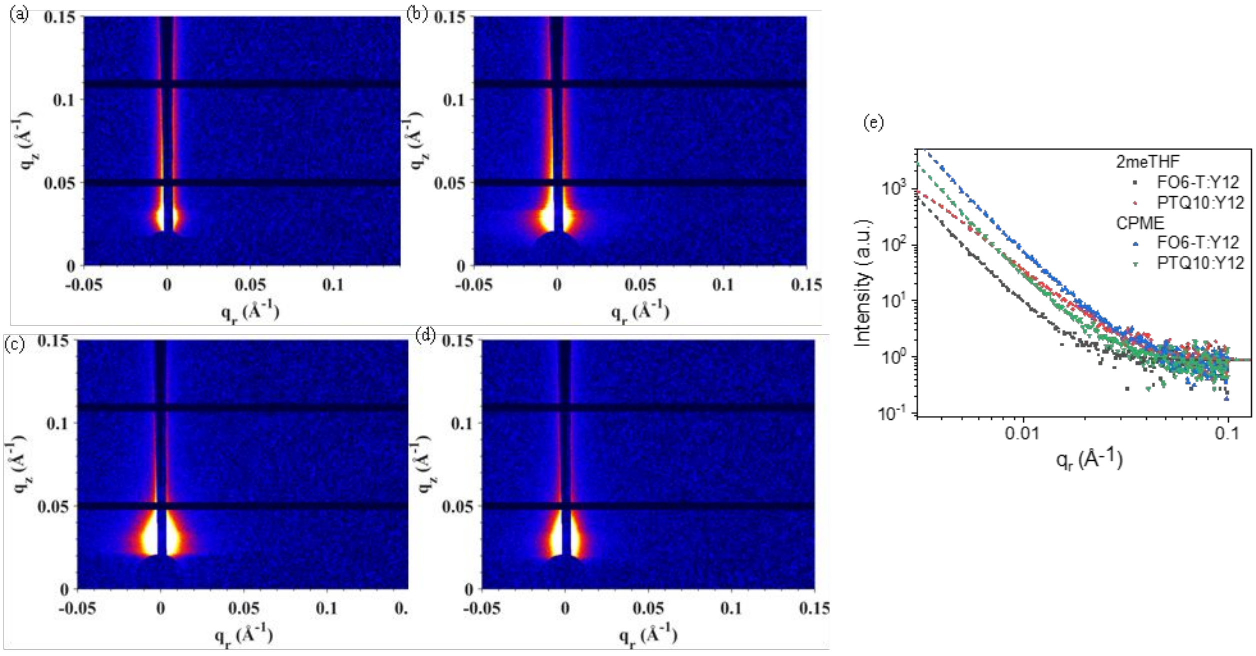

Figure S21: The 2D GISAXS plots of 2MeTHF-processed (a) FO6-T:Y12, (b) PTQ10:Y12 blend films. The corresponding plots for CPME-processed films are shown in (c)-(d). A summary of in-plane line cuts extracted at the Yoneda peak is shown in (e).

Table S10: GIWAXS peak analysis for thin film processed from 2MeTHF.

2MeTHF	q_x lamellar			q_z lamellar 1			q_z lamellar 2			q_z lamellar 3 (very weak)			q_z π-π 1			q_z π-π 2		
	q (Å^{-1})	d (Å)	L_c (Å)	q (Å^{-1})	d (Å)	L_c (Å)	q (Å^{-1})	d (Å)	L_c (Å)	q (Å^{-1})	d (Å)	L_c (Å)	q (Å^{-1})	d (Å)	L_c (Å)	q (Å^{-1})	d (Å)	L_c (Å)
FO6-T	0.28	22.1	67.2	0.30	20.7	96.1	0.59	10.6	50.0	0.86	7.3	-	-	-	-	1.71	3.68	27.3
PTQ10	0.25	25.0	67.7	0.27	23.2	90.5	0.52	12.0	42.7	0.83	7.6	-	1.41	4.44	20.0	1.77	3.55	23.5
Y12	0.34	18.7	28.3	-	-	-	-	-	-	-	-	-	-	-	-	1.76	3.58	16.1
FO6-T: Y12	0.30	21.2	69.8	0.28	22.5	36.6	-	-	-	-	-	-	-	-	-	1.75	3.60	25.5
PTQ10: Y12	0.27	23.3	77.0	0.26	24.2	46.7	-	-	-	-	-	-	-	-	-	1.77	3.55	24.1

Table S11: GIWAXS peak analysis for thin film processed from CPME.

CPME	q_x lamellar 1			q_x lamellar 2			q_z lamellar 1			q_z lamellar 2			q_z lamellar 3 (very weak)			q_z π-π 1			q_z π-π 2		
	q (Å^{-1})	d (Å)	L_c (Å)	q (Å^{-1})	d (Å)	L_c (Å)	q (Å^{-1})	d (Å)	L_c (Å)	q (Å^{-1})	d (Å)	L_c (Å)	q (Å^{-1})	d (Å)	L_c (Å)	q (Å^{-1})	d (Å)	L_c (Å)	q (Å^{-1})	d (Å)	L_c (Å)
FO6-T	0.28	22.1	62.5	-	-	-	0.3	20.5	90.6	0.58	10.8	27.1	0.88	7.2	-	-	-	-	1.71	3.67	17.1
PTQ10	0.21	29.66	258.2	0.27	23.3	137.1	0.27	23.1	96.7	0.52	12.1	52.8	0.6	10.5	121.4	1.53	4.2	37.6	1.87	3.37	39.5
Y12	0.21	29.8	214.7	0.27	23.1	154.6	0.51	12.3	157.9	0.58	10.8	32.1	-	-	-	1.54	4.09	-	1.86	3.37	16.1
FO6-T: Y12	0.21	29.7	258.5	0.28	22.2	90.4	0.29	21.4	217.8	0.53	11.7	150.1	0.6	10.5	66.9	1.49	4.21	15.9	1.76	3.58	24.3
PTQ10: Y12	0.21	30.0	646.6	0.27	23.5	138.4	0.27	23.3	78.1	0.51	12.2	191.7	0.59	10.7	156.5	1.53	4.12	21.9	1.84	3.42	35.1

References

(1) Liu, C.; Zhu, Y.; Chen, J.; Wang, H.; Cao, Y.; Chen, J. Terpinolene Processed PTB7:PC71BM Blend Film for Polymer Solar Cells: A Non-Aromatic and Non-Chlorinated Solvent Predicted by Hansen Solubility Parameters. *Synth Met* **2018**, *242*, 17–22. https://doi.org/10.1016/J.SYNTHMET.2018.05.001.

(2) Sprau, C.; Cruz, A. M.; Bautista, L.; Molina, L.; Wagner, M.; Chochos, C. L.; Pirriera, M. Della; Colsmann, A. Green Inks for the Fabrication of Organic Solar Cells: A Case Study on PBDTTPD:PC61BM Bulk Heterojunctions. *Advanced Energy and Sustainability Research* **2021**, *2* (9), 2100043. https://doi.org/10.1002/AESR.202100043.

(3) Ye, L.; Xiong, Y.; Chen, Z.; Zhang, Q.; Fei, Z.; Henry, R.; Heeney, M.; O'Connor, B. T.; You, W.; Ade, H. Sequential Deposition of Organic Films with Eco-Compatible Solvents Improves Performance and Enables Over 12%-Efficiency Nonfullerene Solar Cells. *Advanced Materials* **2019**, *31* (17), 1808153. https://doi.org/10.1002/ADMA.201808153.

(4) Li, M. J.; Fan, B. B.; Zhong, W. K.; Zeng, Z. M. Y.; Xu, J. K.; Ying, L. Rational Design of Conjugated Polymers for D-Limonene Processed All-Polymer Solar Cells with Small Energy Loss. *Chinese Journal of Polymer Science (English Edition)* **2020**, *38* (8), 791–796. https://doi.org/10.1007/S10118-020-2429-3/METRICS.

(5) Shang, L.; Qu, S.; Deng, Y.; Gao, Y.; Yue, G.; He, S.; Wang, Z.; Wang, Z.; Tan, F. Simple Furan-Based Polymers with the Self-Healing Function Enable Efficient Eco-Friendly Organic Solar Cells with High Stability. *J Mater Chem C Mater* **2022**, *10* (2), 506–516. https://doi.org/10.1039/D1TC05111C.

(6) Nguyen, T. L.; Lee, C.; Kim, H.; Kim, Y.; Lee, W.; Oh, J. H.; Kim, B. J.; Woo, H. Y. Ethanol-Processable, Highly Crystalline Conjugated Polymers for Eco-Friendly Fabrication of Organic Transistors and Solar Cells. *Macromolecules* **2017**, *50* (11), 4415–4424. https://doi.org/10.1021/ACS.MACROMOL.7B00452/SUPPL_FILE/MA7B00452_SI_001.PDF.

(7) Lee, S.; Kim, Y.; Wu, Z.; Lee, C.; Oh, S. J.; Luan, N. T.; Lee, J.; Jeong, D.; Zhang, K.; Huang, F.; Kim, T.-S.; Woo, H. Y.; Kim, B. J. Aqueous-Soluble Naphthalene Diimide-Based Polymer Acceptors for Efficient and Air-Stable All-Polymer Solar Cells. *ACS Appl Mater Interfaces* **2019**, *11* (48), 45038–45047. https://doi.org/10.1021/acsami.9b13812.

(8) Fan, B.; Ying, L.; Zhu, P.; Pan, F.; Liu, F.; Chen, J.; Huang, F.; Cao, Y. All-Polymer Solar Cells Based on a Conjugated Polymer Containing Siloxane-Functionalized Side Chains with Efficiency over 10%. *Advanced Materials* **2017**, *29* (47), 1703906. https://doi.org/10.1002/ADMA.201703906.

(9) Zhu, C.; Li, Z.; Zhong, W.; Peng, F.; Zeng, Z.; Ying, L.; Huang, F.; Cao, Y. Constructing a New Polymer Acceptor Enabled Non-Halogenated Solvent-Processed All-Polymer Solar Cell with an Efficiency of 13.8%. *Chemical Communications* **2021**, *57* (7), 935–938. https://doi.org/10.1039/D0CC07213C.

(10) Ye, L.; Li, W.; Guo, X.; Zhang, M.; Ade, H. Polymer Side-Chain Variation Induces Microstructural Disparity in Nonfullerene Solar Cells. *Chemistry of Materials* **2019**, *31* (17), 6568–6577. https://doi.org/10.1021/acs.chemmater.9b00174.

(11) Gasparini, N.; Paleti, S. H. K.; Bertrandie, J.; Cai, G.; Zhang, G.; Wadsworth, A.; Lu, X.; Yip, H. L.; McCulloch, I.; Baran, D. Exploiting Ternary Blends for Improved Photostability in High-Efficiency Organic Solar Cells. *ACS Energy Lett* **2020**, *5* (5), 1371–1379. https://doi.org/10.1021/acsenergylett.0c00604.

(12) Gasparini, N.; Salvador, M.; Fladischer, S.; Katsouras, A.; Avgeropoulos, A.; Spiecker, E.; Chochos, C. L.; Brabec, C. J.; Ameri, T. An Alternative Strategy to Adjust the Recombination Mechanism of Organic Photovoltaics by Implementing Ternary Compounds. *Adv Energy Mater* **2015**, *5* (24), 1–7. https://doi.org/10.1002/aenm.201501527.
